# Targeted splice sequencing reveals RNA toxicity and therapeutic response in myotonic dystrophy

**DOI:** 10.1093/nar/gkab022

**Published:** 2021-01-27

**Authors:** Matthew K Tanner, Zhenzhi Tang, Charles A Thornton

**Affiliations:** Medical Scientist Training Program, University of Rochester Medical Center, Rochester, NY 14642, USA; Department of Neurology, University of Rochester Medical Center, Rochester, NY 14642, USA; Department of Neurology, University of Rochester Medical Center, Rochester, NY 14642, USA

## Abstract

Biomarker-driven trials hold promise for therapeutic development in chronic diseases, such as muscular dystrophy. Myotonic dystrophy type 1 (DM1) involves RNA toxicity, where transcripts containing expanded CUG-repeats (CUG^exp^) accumulate in nuclear foci and sequester splicing factors in the Muscleblind-like (Mbnl) family. Oligonucleotide therapies to mitigate RNA toxicity have emerged but reliable measures of target engagement are needed. Here we examined muscle transcriptomes in mouse models of DM1 and found that CUG^exp^ expression or *Mbnl* gene deletion cause similar dysregulation of alternative splicing. We selected 35 dysregulated exons for further study by targeted RNA sequencing. Across a spectrum of mouse models, the individual splice events and a composite index derived from all events showed a graded response to decrements of Mbnl or increments of CUG^exp^. Antisense oligonucleotides caused prompt reduction of CUG^exp^ RNA and parallel correction of the splicing index, followed by subsequent elimination of myotonia. These results suggest that targeted splice sequencing may provide a sensitive and reliable way to assess therapeutic impact in DM1.

## INTRODUCTION

Myotonic dystrophy type 1 (DM1) is a dominantly inherited neuromuscular disease caused by expansion of a CTG trinucleotide repeat in the 3′ untranslated region of *DMPK* (1). The disease mechanism involves RNA dominance, in which transcripts from the mutant allele acquire a deleterious gain-of-function (2). The mutant transcripts contain expanded CUG-repeats (CUG^exp^) and form nuclear foci in muscle, cardiac and CNS cells (3–5).

Muscleblind-like (MBNL) splicing factors were implicated in DM1 pathogenesis through screens for nuclear proteins that bind to CUG^exp^ RNA (6). Few RNA binding proteins (RBPs) actually exhibited this property, of which MBNL1 was highly predominant. MBNL1 and MBNL2 are both sequestered in RNA foci in DM1 cells (4,7,8). The resulting dysregulation of alternative splicing contributes to core symptoms of the disease, such as insulin resistance, myotonia, muscle weakness, and cardiac arrhythmia (9–15). MBNL proteins are highly conserved and tightly regulated, and mice with *Mbnl1/Mbnl2* deletion exhibit cardinal features of DM1, such as myotonia, myopathy and heart block (16,17).

Efforts to treat DM1 by reducing the synthesis of CUG^exp^ RNA, accelerating its decay, blocking its interactions with binding proteins, or increasing the expression of Mbnl have all shown benefit in animal models, suggesting that RNA dominant mechanisms are unusually tractable for therapeutic intervention (reviewed in reference (18)). Translation to DM1 patients, however, has not yet been accomplished.

Biomarkers are useful tools for drug development, especially for chronic diseases in which the clinical response is slow or incomplete. Biomarker evidence is useful at all steps of drug development, from preclinical studies to pivotal trials and drug approval. Although major effort has been directed towards targeting the mutant *DMPK* mRNA in DM1, successful engagement of this target may be difficult to assess in clinical trials. One limitation is that long CUG^exp^ RNAs are difficult to extract from cells and tissue (3), a property shared with other long nuclear RNAs that are highly structured and protein-bound (19). Another challenge is that mutant and wild-type (WT) *DMPK* transcripts reside in different cellular compartments (3) and may respond differently to treatment (20) yet are difficult to discriminate in assays. Furthermore, expanded DM1 repeats are highly unstable in somatic cells, generating long expansions that are extremely heterogeneous in muscle tissue (21). Accordingly, *DMPK* transcript levels cannot be equated to CUG^exp^ load. Finally, the extent of CUG^exp^ reduction or Mbnl release required to restore splicing regulatory activity is presently unknown and may differ between individuals.

Here, we used RNAseq to examine mouse models and found that effects of CUG^exp^ expression or *Mbnl* deletion on the muscle transcriptome were highly concordant. We selected 35 DM1-affected splice events for further analysis by targeted RNA sequencing. The selected splice events showed strong developmental regulation in wildtype (WT) mice, and in adult DM1 mice they reverted to fetal splicing patterns. Unexpectedly, a subset of splicing changes in DM1 mice could be attributed to myotonia as an indirect consequence of Mbnl loss. A composite index derived from all 35 splice events showed an incremental response to progressive inactivation of *Mbnl* alleles or stepwise accumulation of CUG^exp^ RNA. Conversely, treating CUG^exp^-expressing mice with antisense oligonucleotides (ASOs) caused a reduction of toxic RNA and parallel correction of the splicing index, followed by rescue of myotonia. Taken together, these results indicate that targeted RNA splice sequencing provides a reliable indicator of RNA toxicity that responds rapidly to treatment.

## MATERIALS AND METHODS

### Mouse lines

*Mbnl1* null mice having targeted deletion of exon 3 were previously described (16). Mice with disruption of *Mbnl2* by integration of a gene trap vector were previously described (8). HSALR transgenic mice in line 20b express a human *ACTA1* transgene with about 220 CTG repeats in the 3′ UTR. The HSALR transgene is highly expressed in skeletal muscle (22). Similar mice having about 440 CTG repeats in an *ACTA1* transgene, called HSAXLR transgenic mice, were derived using PhiC31 integrase as previously described (23). Mice in founder line HSAXLRe have a multi-copy transgene integration and high expression. DMSXL transgenic mice were obtained from Dr Geneviève Gourdon and carry a human *DMPK* transgene with ∼1200 CTG repeats (24). *adr-mto2J* having recessive generalized myotonia and a frameshift mutation in the *Clcn1* muscle-specific chloride channel (25) were obtained from Jackson Labs. All lines mentioned above were derived or extensively backcrossed (>12 generations) onto the FVB/N (Taconic) background. *mdx* mice on the C57BL/10ScSn background were obtained from Jackson Labs. The age and sex of each animal in the study is listed in Supplemental Data Tables S4 and S5.

### RNA isolation

Mouse tissues were flash frozen in liquid nitrogen and stored at –80°C. RNA was isolated as previously described using Tri-Reagent (Sigma-Aldrich), except that homogenization was carried out using Bullet Blender (Next Advance). RNA was re-isolated on RNeasy columns (Qiagen) with on-column DNase I-treatment.

### Conventional RNAseq and analysis

Quadriceps muscle was harvested from WT mice, homozygous HSALR20b mice, and mice with homozygous deletion in *Mbnl1* and heterozygous disruption of *Mbnl2* (Mbnl¾KO mice). Tissue was obtained from mice between 5 and 10 weeks of age, with two males and two females per group. Libraries were prepared from polyA-selected RNA using TruSeq Stranded mRNA Library Prep Kit (Illumina) and sequenced on Illumina HiSeq 2500, generating an average of 59 million paired reads (125 bp) per sample. Raw reads were quality-filtered and trimmed using Trimmomatic v0.36 and aligned to the mm10 mouse reference genome using HISAT2 v2.0.4 (26,27). Transcript abundance estimation was performed using Stringtie v1.3.2 and differential gene expression was performed using DESeq2 v1.16.1 (28,29). rMATS v3.2.5 was used for differential analysis of alternative splicing (30).

### Motif enrichment analysis

RNA sequences flanking exons that showed misregulated alternative splicing in DM1 models ($| {\Delta PSI} |$$ \ge$ 0.05, FDR-adjusted *P* value < 0.05) were analyzed for local enrichment of all 5-mers, compared to a background set of alternatively spliced exons (PSI between 0.1 and 0.9 in WT mice) that were not misregulated in DM1 models. For this analysis we examined 350 nt of flanking intron and 50 nt of adjacent exon. Only pentamers with moderate-to-strong interspecies conservation (average base-wise phyloP $ \ge \;$0.5 for Euarchontoglires subset) were retained for enrichment analysis. Significance of local enrichment for each pentamer at each position was derived by Fisher exact test with Benjamini-Hochberg (BH) correction. Enrichment and significance calculations utilized 50 nt sliding windows. To test for predictive capacity of all pentamers, we counted conserved pentamer occurrences in upstream and downstream flanking sequences (200 nt of intron and 50 nt of adjacent exon on either side) to construct a linear model for each pentamer using pentamer counts as explanatory variables and ΔPSI as the output variable. We then ranked all pentamers by adjusted coefficient of determination (*R*^2^).

### Design of targeted splice sequencing library

From the deep RNAseq datasets, cassette exon splice events were rank-ordered by absolute size of the PSI shift in DM1 versus WT mice. The 23 events having largest effects and gene structure amenable to amplicon sequencing were selected for targeted analysis. We also included 12 exons that showed splicing misregulation in the RNAseq datasets and which were previously identified as mis-spliced in DM1 patients (*Atp2a1*, *Best3*, *Bin1*, *Clasp1*, *Clcn1*, *Kif13a*, *Mbnl1*, *Mbnl2*, *Nfix*, *Opa1*, *Ryr1* and *Vps39*), including several events implicated in muscle dysfunction. One splice event in *Brd2* was included as negative control, which showed no affect in DM1 models. Primers were designed to avoid amplification across more than one alternatively spliced exon. To reduce any detection bias that may result from preferential amplification of shorter splice products, we sought to minimize the proportional size difference between exon-inclusion versus exon-exclusion amplicons. On average, the cassette exons comprised 14 ± 11% (mean ± standard deviation) of total amplicon length. We also optimized amplicon sizes for cluster generation on Illumina sequencers, aiming to generate amplicons of at least 400 bp when possible (mean 617 ± 87 bp standard deviation). For each amplicon, either the forward or reverse primer was less than 125 bp from the cassette exon to ensure that informative splice junctions were captured with high quality base calls. To obtain rough balance of sequence reads across 36 transcripts despite a 4000-fold range of expression levels, the 36 primer sets were distributed across 4 multiplex first-stage PCR reactions (PCR1) of 17, 15, 13 and 12 cycles (PCR1 A, B, C and D respectively), based on transcript abundance and avoidance of primer–dimers. For some amplicons, primer concentration in PCR1 was reduced from 200 to 50 nM to further reduce over-representation of highly expressed transcripts. All PCR1 primers included a 5′ adapter sequence for incorporation of sample barcodes and priming sites for Illumina sequencing in a second-stage PCR (PCR2). Amplicons and primers are described in Supplemental Data Table S3.

### Targeted RNAseq library preparation

For each sample, 200 ng of RNA was reverse transcribed using anchored oligo(dT)18 (IDT) and SuperScript II (Invitrogen) in 20 μl total volume according to the manufacturer's protocol. For each cDNA, four multiplex PCR1 reactions were carried out using Q5 High-Fidelity Polymerase (NEB) and 1 μl cDNA as input. PCR1 annealing temperature was 70°C and extension time was 55 s. PCR1 reactions were purified on AMPure XP beads (Beckman Coulter) using a 1:1 sample-to-bead ratio after combining all of PCR1 reactions A, B and C with ¼ of reaction D. Purified PCR1 amplicons were eluted in 40μl, of which 15 μl was used as input for PCR2, consisting of 12 cycles of adapter incorporation and sample barcoding using Nextera XT Index Kit (FC-131-1001, Illumina) primers and Q5 Polymerase. PCR2 annealing temperature was 65°C and extension time was 1 min. PCR2 products were purified on DNA Clean & Concentrator-5 columns (Zymo Research) or AMPure XP beads, and library concentration was quantified using Qubit dsDNA BR assay (Invitrogen). Up to 24 sample libraries were combined in equal amounts by DNA mass and purified on AMPure XP beads with a 1:1 sample-to-bead ratio. Purified amplicon pools were used for 150 bp paired-end sequencing on MiSeq with MiSeq v2 Micro reagent kits (Illumina), producing ∼4 million total paired reads per run. Using pools of 24 samples per run, we obtained an average of 165 000 read pairs per sample and 4100 isoform-specific reads per splice event from adult quadriceps samples (*n* = 156 mice).

### Quantification of alternative splicing from targeted RNAseq data

Reads were aligned to reference sequences of inclusion and exclusion splice isoforms, provided in the Supplemental Sequences File, using HISAT2 v2.1.0 with no-softclip and no-spliced-alignment flags. Primary aligned reads spanning splice junctions were subsetted from the resulting bam files using samtools v1.7. Isoform-specific reads with unambiguous alignments were then counted, and PSI for each event was calculated as the fraction of inclusion-isoform reads relative to all inclusion- and exclusion-isoform reads. Mouse DM1 Splicing Index (mDSI) was calculated as follows. For each sample *i*, normalized splicing values were calculated for each splice event *j* as (PSI*_i,j_* – PSI_wildtype,*j*_)/(PSI_DM95,*j*_ – PSI_wildtype,*j*_) where PSI_wildtype,*j*_ is the median PSI for event *j* across nine WT mice, and PSI_DM95,*j*_ is the 95th percentile for most severely affected PSI in DM1 models. For these calculations, the 95th percentile for DM1 models was determined from the 97 DM1 model mice shown in Supplemental Figure S7, comprising all *Mbnl* gene deletion, HSALR, and HSAXLRe mice, including crosses. mDSI is then calculated as the mean of all normalized splicing values except *Brd2*, the negative control.

### Western blots

Muscle tissue was homogenized by mortar and pestle in RIPA Lysis and Extraction Buffer (Thermo Scientific) with 1× Halt Protease Inhibitor Cocktail (Thermo Scientific) and 0.05 U/μl Benzonase (Sigma Aldrich) and protein concentration was quantified by Pierce BCA Protein Assay Kit (Thermo Scientific). Up to 30 μg protein was denatured in Novex Tris–glycine SDS sample buffer (2×) (Invitrogen) for 5 min at 85°C. NuPAGE Sample Reducing Agent (Invitrogen) was added to 1× final concentration prior to separating protein on Novex 4–20% Tris–glycine Mini Gel (Invitrogen) and transferring to nitrocellulose membrane (LI-COR) according to manufacturer's protocol. Following 1 h of blocking with Intercept (PBS) Blocking Buffer (LI-COR) at room temperature, membrane was incubated overnight at 4°C in blocking buffer with 0.2% Tween-20 and mouse monoclonal antibody to α-tubulin (clone DM1A, Abcam ab7291) at 1:10 000 dilution and rabbit polyclonal antibody to Mbnl1 (A2764) (8) at 1:10 000 dilution. Protein detection was performed using IRDye 680RD Goat anti-Rabbit IgG and IRDye 800CW Goat anti-Mouse IgG at 1:15 000 each with imaging on Odyssey 9120 imager (LI-COR). Band intensity quantification was performed using Image Studio Lite software (LI-COR).

### Antisense oligonucleotide treatment of HSALR mice and electromyography

ASOs (courtesy of F. Rigo and F. Bennett, Ionis Pharmaceuticals) were conjugated to palmitic acid (C16) *via* a hexylamino linker (HA) at the 5′ end of the ASO. ASO Ionis 992948 is the conjugated version of Ionis 445236, a gapmer targeting *ACTA1* transgene mRNA, consisting of 10 central 2′-deoxyribonucleotides flanked on either side by five 2′-*O*-methoxyethyl-modified nucleotides (MOE), with phosphorothioate linkages throughout, as previously described (31,32). All cytosine residues were 5-methylcytosine. ASO conjugate 1047143 is a non-targeting control. ASO sequences are as follows: 5′-C16-HA-P_o_-CCATTTTCTTCCACAGGGCT-3′ (992948) and 5′-C16-HA-P_o_-CCTTCCCTGAAGGTTCCTCC-3′ (1047143). Active and control ASOs were dissolved in phosphate-buffered saline (PBS) and all treatments were delivered by subcutaneous injection in the interscapular region. For the time-course experiment, homozygous HSALR mice were treated with 25 mg/kg of active or control ASO twice weekly for 3–28 days. For the single-injection experiment, homozygous HSALR mice were treated with a single injection of 12.5, 25, 50 or 100 mg/kg of active ASO, or 100 mg/kg of control ASO, and sacrificed 10 days later. Immediately prior to sacrifice, mice were anesthetized with isoflurane and electromyography (EMG) was performed using bipolar needle electrodes as previously described (31). Myotonia was scored on a 0–3 scale, corresponding to the frequency of myotonic discharges evoked by 10–20 advances of the needle electrode per muscle: 3, 100% of insertions; 2, >50% of insertions; 1, <50% of insertions; 0.5, almost no insertions; 0, no insertions.

### Quantitative RT-PCR (RT-qPCR)

Quadriceps muscle RNA (300 ng) was reverse transcribed using random primers (Invitrogen) and SuperScript II in 20 μl total volume according to the manufacturer's protocol. qPCR reactions were carried out using TaqMan Fast Advanced Master Mix (Applied Biosystems) and 2 μl input cDNA. For *ACTA1* qPCR, forward primer was GTAGCTACCCGCCCAGAAACT, reverse primer was CCAGGCCGGAGCCATT and custom TaqMan probe was ACCACCGCCCTCGTGTGCG (3′ MGBNFQ quencher and 5′ 6FAM dye, Applied Biosystems). For *Gtf2b* reference qPCR, Gene Expression Assay Mm00663250_m1 (4331182, Applied Biosystems) was used. qPCR was performed on Applied Biosystems StepOne using fast ramp speed. *ACTA1* transgene expression relative to *Gtf2b* was calculated using the ΔΔC_t_ method.

### Statistical analysis

Statistical tests and modeling, including linear modeling, t tests (two-sided unless otherwise stated), derivation of coefficients of determination, *F*-tests, Fisher exact tests, principal component analysis, and tSNE analysis were performed in R. Model fitting for the generation of four-parameter logistic regressions was performed using the minpack.lm package in R. tSNEs were generated using the Rtsne package in R. Multiple testing correction was performed using the Benjamini-Hochberg method. Gene ontology term enrichment and transcription factor motif enrichment were generated using Enrichr (33).

## RESULTS

### Concordant effects of CUG^exp^ expression or *Mbnl* deletion on the muscle transcriptome

To obtain a comprehensive view of splicing regulation in mouse DM1 models we used RNAseq to examine quadriceps muscle from human skeletal actin long-repeat (HSALR) transgenic mice and *Mbnl1*^−/−^/*Mbnl2*^+/−^ mice, designated here as Mbnl¾KO mice. HSALR mice express sarcomeric actin (*ACTA1*) mRNA with ∼220 CUG repeats in the 3′ untranslated region (22). Consistent with previous reports, through breeding of *Mbnl1*^+/−^/*Mbnl2*^+/−^ knockout mice we were unable to recover double homozygous knockouts at weaning (17). We therefore examined Mbnl¾KO mice, the highest constitutive reduction of Mbnl compatible with post-weaning survival. We used rMATS (30) to analyze alternative splicing in each model compared to WT mice of the same strain background.

**Figure 1.**
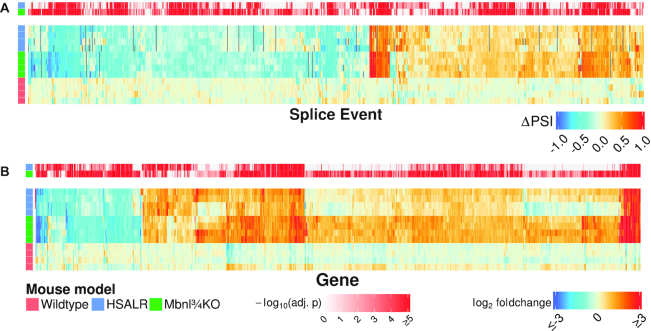
Concordant effects of CUG^exp^ expression or Mbnl loss on alternative splicing (A) and gene expression (B) in quadriceps muscle. Heat maps show transcriptome changes in HSALR transgenic mice (top four rows of each map) or Mbnl¾KO mice (middle four rows) as compared to WT controls (bottom four rows). (**A**) Heat map of 752 cassette exon splice events that were misregulated in either DM1 model versus WT with |ΔPSI| $ \ge$ 0.20 and FDR-adjusted *P* < 0.05. (**B**) heat map of 877 genes that were differentially expressed at least 2-fold in either DM1 model versus WT, with Benjamini-Hochberg (BH) adjusted *P* < 0.05. *P* values for comparison to WT are visually displayed above each heat map. Changes not reaching statistical significance in one group are indicated in gray. Two males and two females were analyzed per group, ages 5–10 weeks.

The largest group of alternative splice events affected by CUG^exp^ expression or *Mbnl* deletion were cassette exons (72 or 71% of splicing changes, respectively) (Supplemental Figure S1a and Supplemental Data Table S1). For both models, we quantified splicing of cassette exons as the absolute difference of percent-spliced-in (ΔPSI) relative to WT controls. Using threshold cutoffs of |ΔPSI| ≥ 20% and false discovery rate (FDR)-adjusted *P* < 0.05, 752 cassette exons were misregulated in one or both models, and most were affected in the same direction in both (Figure 1a). Among all 2070 cassette exons that were misregulated to any extent in either model (FDR-adjusted *P* < 0.05, no threshold for ΔPSI), 93% were concordantly misregulated in both models (Supplemental Figure S1b). The extent of splicing alteration was highly correlated between models (*R*^2^ = 0.75) but tended to be greater in magnitude in Mbnl¾KO mice.

**Figure 2.**
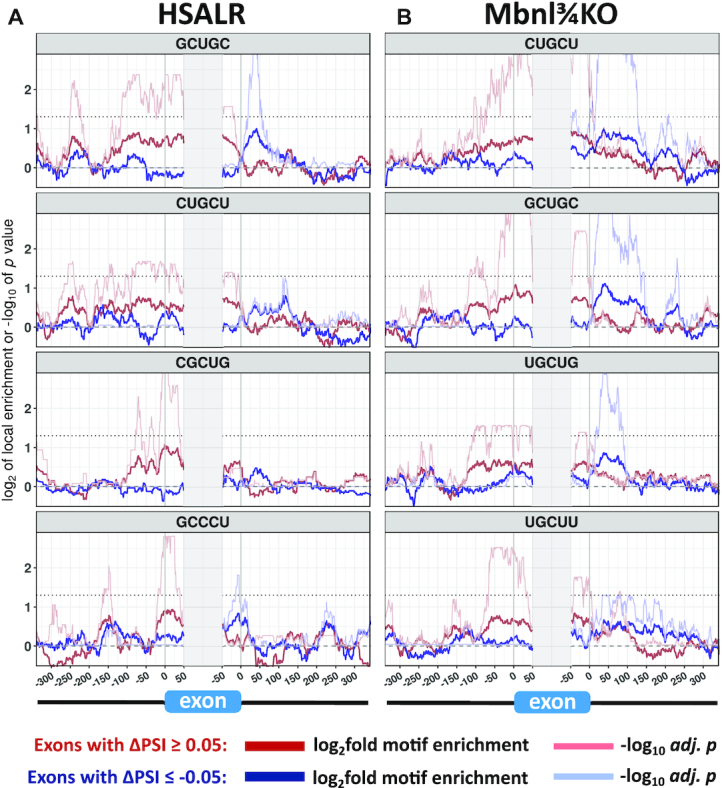
Enrichment of Mbnl-binding motifs in introns flanking exons that show misregulated alternative splicing in DM1 models. The sequences flanking misregulated alternative exons (|ΔPSI| $ \ge$ 0.05 and *P* < 0.05) in HSALR (**A**, 1005 exons) or Mbnl¾KO mice (**B**, 1506 exons) were probed for local enrichment of all pentamers relative to a background set of alternative exons (0.10 $ \le$ PSI $ \le$ 0.90) whose splicing was not affected in DM1 models (1886 exons for HSALR and 1667 exons for Mbnl¾KO). The four pentamers having most significant enrichment, each of which corresponds to a potential Mbnl binding site, are shown for each model with blue traces for exons that have increased inclusion in DM1 models and red traces for exons that have decreased inclusion. Thick blue and red traces indicate fold-enrichment of the indicated pentamer using a 50nt sliding window, and thin blue and red traces indicate −log_10_ of the *P* value for enrichment (Fisher exact test with BH correction for multiple testing). The horizontal dotted line represents threshold for significance (−log_10_ of 0.05).

To gauge direct involvement of Mbnl and test for other factors that may contribute to splicing changes, we tested for enrichment of conserved pentamer sequence motifs in introns flanking the dysregulated exons, as compared to a control set of alternatively spliced but DM1-unaffected exons. In both mouse models the pentamers showing the most significant enrichment were Mbnl-binding motifs, comprising GC dinucleotides embedded in pyrimidines (Figure 2) (34,35). The location of motifs fit with previously described patterns for Mbnl regulation (34,36,37), in which binding motifs in downstream introns were associated with increased exon skipping in DM1 models and motifs in upstream introns or exon bodies were associated with increased exon inclusion. Splicing outcomes were also predicted by the multiplicity of Mbnl motifs in these positions, supporting a mechanism in which multivalent binding drives stronger splicing regulation (Supplemental Figure S2 and Supplemental Tables S1 and S2) (38). Of all pentamers, only one non-Mbnl binding motif (CUAAU and its variant UAAUC) was a significant predictor of splicing outcomes, which may indicate a role for Quaking protein in splicing dysregulation (discussed below) (39).

These results confirmed at transcriptome-wide scale the impression that nearly all effects on splicing regulation in the HSALR model can be explained by loss of Mbnl function (36). However, effects of CUG^exp^ expression or Mbnl loss on the muscle transcriptome extend well beyond alternative splicing. Consistent with previous work using microarrays (36,40) RNAseq showed major changes of gene expression in DM1 models (Supplemental Data Table S2). Again, a high proportion of changes (81%) were concordant in HSALR and Mbnl¾KO mice (Figure 1B and Supplemental Figure S1c). Gene ontology analysis indicated that genes upregulated in Mbnl¾KO mice were enriched for functional categories of extracellular matrix organization, cytoskeleton organization, and cell migration, whereas downregulated genes were enriched for carbohydrate metabolism and muscle contraction (Supplemental Table S3). Among the downregulated genes, analysis of promoter regions for transcription factor binding motifs showed significant enrichment of cognate elements for the myocyte enhancer factor 2 (Mef2) family (Supplemental Table S4). Of note, splicing changes may underlie this effect, because WT mice expressed mainly the α_2_ splice isoforms of *Mef2c* and *Mef2d*, which act cooperatively with Myog and Myod1 to promote myogenic differentiation and sustain muscle gene expression (41,42), whereas DM1 models expressed higher levels of the α_1_ isoforms (Supplemental Figure S3), which antagonize α_2_ isoforms and interact with inhibitory class II histone deacetylases that repress gene expression (43–47).

### Targeted *vs*. non-targeted RNAseq

While RNAseq is a powerful discovery tool for alternative splicing, ultra-deep sequencing is required for precise quantification of splicing for low-abundance transcripts. However, if the key alterations are conveyed by a subset of splice events, then targeted sequencing is potentially a more efficient tool to assess splicing misregulation. To test this possibility we used multiplexed RT-PCR followed by amplicon sequencing for targeted analysis of DM1-affected splice events. We incorporated multiple splice events in our targeted panel, since exons respond differently to marginal changes of Mbnl expression or DM1 severity (21,48,49). Furthermore, statistical modeling has shown that estimates of Mbnl activity derived from splicing outcomes have improved accuracy when multiple splice events are included, up to a maximum of around 30 events (48). Accordingly, we chose 35 cassette exons that showed major changes of alternative splicing in the HSALR and Mbnl¾KO RNAseq datasets, including 23 selected for having large shifts of PSI, and another 12 selected because they were previously shown to exhibit splicing misregulation in DM1 patients (*Atp2a1*, *Best3*, *Bin1*, *Clasp1*, *Clcn1*, *Kif13a*, *Mbnl1*, *Mbnl2*, *Nfix*, *Opa1*, *Ryr1* and *Vps39*), including several that impact muscle function. For example, skipping of the 15 nt exon of *Ryr1* causes increased calcium release from the sarcoplasmic reticulum (50), inclusion of the 79 nt exon of *Clcn1* eliminates ion conductance of the muscle-specific chloride channel (51), and skipping of the 45 nt exon of *Bin1* inhibits formation of transverse tubules (12). As a negative control we also included the 92 nt exon of *Brd2*, which is alternatively spliced in muscle but not affected in DM1 models, for a total of 36 splice events.

Aside from the simplicity of library preparation and data analysis, a key advantage of targeted splice sequencing is increased efficiency, generating more reads of informative splice junctions and ultimately higher precision. For example, >90% of targeted reads were informative about a splice event of interest compared to 0.002% of non-targeted reads. A potential disadvantage, however, is detection bias. If shorter splice products are amplified with greater efficiency, then exon-skipping isoforms will be over-represented in the amplicon pools. To test for this bias we compared targeted *vs* conventional RNAseq for the WT, HSALR, and Mbnl¾KO datasets described above. Targeted RNAseq did indeed exhibit detection bias, but the effect was generally small, and not all splice events were affected (Supplemental Figure S4a). The mean under-detection of exon inclusion was –4.4% PSI across all events and samples. As expected, detection bias was greater for longer cassette exons, where the size difference of inclusion versus skipped isoforms was most pronounced. The largest alternative exons in the panel (*Fn1* at 270 nt, *Trim55* at 288 nt and *Ttn* at 303 nt) produced under-detection of exon inclusion by –8%, –11% and –7%, respectively (Supplemental Figure S4b). However, detection bias was not solely a matter of exon size. The transcripts showing greatest discrepancy were *Mbnl2* and the negative control, *Brd2*, where under-detection of exon inclusion by targeted RNAseq was –21% and –18%, respectively, despite relatively small size of the alternative exons (95 nt and 92 nt). On further inspection of non-targeted RNAseq for these transcripts, it was apparent that some of the exon-inclusion isoforms showed retention of the upstream intron (9% and 19%, respectively), producing splice products that were detected by conventional RNAseq but prohibitively large for targeted RNAseq. Taken together, these results supported the feasibility of using targeted sequencing to assess splicing for a panel of alternative exons, with the caveat that large cassette exons show modest under-detection and that intron retention is overlooked. However, for our purpose these biases were not limiting and were more than compensated by 30-fold higher average read counts per splice event, greater uniformity of read coverage across events, and narrower confidence limits for the PSI estimate despite ∼350-fold lower total sequencing depth per sample. Furthermore, by principal component analysis of conventional RNAseq data for 1148 misregulated exons, we found that splicing variance among WT, HSALR and Mbnl¾KO mice was dominated by the first principle component, accounting for 55% of between-mouse variance (Supplemental Figure S5a). Targeted splice sequencing recapitulated nearly all of the group differences in this principle component (*R*^2^ = 0.99, Supplemental Figure S5c), suggesting that the 35 events we selected have captured the main source of disease-related splicing variance.

### Developmental regulation of exons with misregulated alternative splicing in DM1

**Figure 3.**
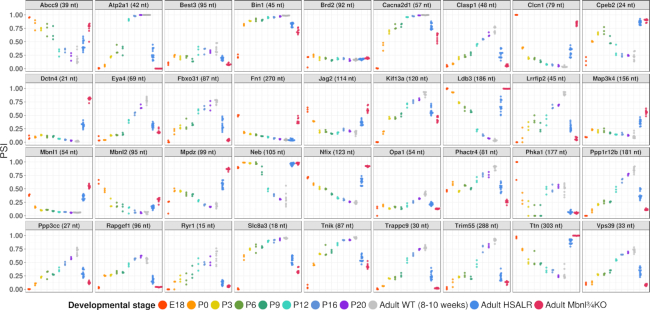
DM1-affected alternative exons are developmentally regulated in wildtype mice. Targeted splice sequencing of hindlimb tissue (E18 and P0) or quadriceps muscle (P3 through adult) in WT mice, as compared to adult quadriceps from HSALR and Mbnl¾KO mice.

Several exons that are misregulated in DM1 have been shown to undergo developmental regulation in WT mice, which mainly occurred in the interval between post-natal day 2 (P2) and P20, a period of intensive muscle growth and remodeling (8). To determine whether regulation in this interval is a general feature of DM1-affected splice events, we performed targeted splice sequencing of WT mice at intervals from embryonic day 18 (E18) to maturity (8–10 weeks). The results showed large developmental transitions for nearly all events on our targeted panel (Figure 3). Consistent with previous data, many splice events showed abrupt shifts of exon inclusion between P6 and P16. Other events, however, showed gradual shifts throughout the entire interval from E18 to P20 (*Clasp1*, *Nfix* and *Ppp3cc*), or abrupt transitions between E18 and P0 (*Bin1*, *Clcn1*, *Fn1*, and *Mbnl1*). A few events showed more complex developmental shifts (e.g. *Cpeb2* and *Mpdz*), or complete absence of post-E18 regulation (the 21 nt exon of *Dctn4* and the 156 nt exon of *Map3k4*). We also examined diaphragm muscle from P0 through P20 and observed broadly similar patterns (Supplemental Data Table S5). In contrast, the negative control exon, in *Brd2*, showed stable inclusion throughout development in both muscles. Taken together with previous studies (8,52,53), these results confirm that splicing changes in DM1 mainly reflect reversion to fetal or neonatal patterns.

### Variations of alternative splicing among muscles

**Figure 4.**
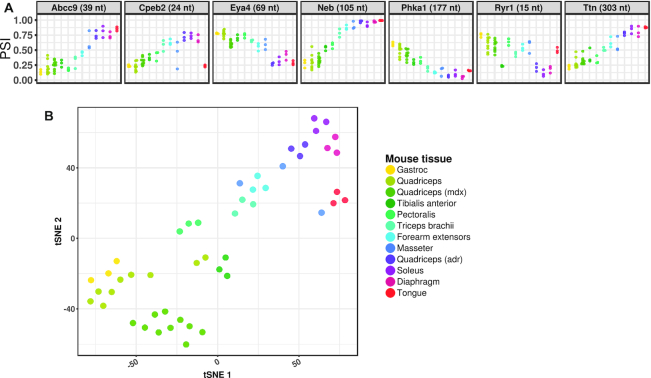
A subset of DM1-affected splice events show differential regulation among muscles. (**A**) Targeted splice sequencing of 10 muscles in adult WT mice showing differential regulation of alternative splicing for seven splice events. (**B**) *t*-distributed stochastic neighbor embedding (tSNE) analysis showed divergence of splicing patterns between muscle groups that are predominantly fast-twitch (gastrocnemius, quadriceps, and tibialis anterior) or slow-twitch (soleus and diaphragm). Splicing patterns in myotonic *adr* quadriceps most closely resembled WT soleus and diaphragm, while splicing in dystrophic *mdx* quadriceps was similar to WT quadriceps.

DM1 is characterized by preferential involvement of distal muscles of the limbs, raising the possibility that vulnerable muscles have higher CUG^exp^ expression or lower MBNL activity. To evaluate the latter possibility we used targeted splice sequencing to examine ten different muscles from WT mice (Supplemental Figure S6). We found minimal splicing differences among muscles for most exons, including proven direct targets of Mbnl regulation, such as *Atp2a1*, *Bin1*, *Ldb3*, *Nfix* and *Mbnl1* (12,38,48,54,55). However, seven of the 36 events—*Abcc9*, *Cpeb2*, *Eya4*, *Neb*, *Phka1*, *Ryr1* and *Ttn*—showed striking differences among muscles (Figure 4A). For these seven events, the splicing outcomes in WT soleus and diaphragm were substantially different from WT quadriceps and instead resembled splicing in quadriceps from Mbnl¾KO mice. Analysis by *t*-distributed stochastic neighbor embedding (tSNE) suggested that splicing in WT mice diverged along an axis of fiber type predominance, from fast-twitch glycolytic (gastrocnemius and quadriceps) to fast- or slow-twitch oxidative fibers (masseter, diaphragm, and soleus) (Figure 4B) (56,57). Taken together, these results suggested that basal levels of Mbnl activity are generally similar across muscles but that a subset of DM1-affected exons exhibit muscle-specific regulation in WT mice, likely reflecting the effects of fiber type specification on alternative splicing.

### Contribution of muscle regeneration or myotonia to splicing changes

*Mdx* mice, a model for Duchenne muscular dystrophy, were previously shown to have modest changes of alternative splicing, attributed to muscle regeneration (58). Accordingly, we wanted to determine which events on our panel may exhibit a non-specific response to chronic myopathy or regeneration, as exemplified in *mdx* mice. We examined quadriceps muscle from 11- to 15-week-old *mdx* mice, when muscles are known to exhibit focal necrosis, inflammation and central nuclei (59,60). Only four events in *mdx* mice differed in exon inclusion from WT by greater than 10% (*Clasp1*, *Eya4*, *Opa1* and *Trim55*), and none approached the severity of splicing misregulation in HSALR or Mbnl¾KO mice (Supplemental Figure S6). Furthermore, *mdx* quadriceps clustered near WT quadriceps on tSNE analysis (Figure 4B).

Studies of excitable cells, such as cortical neurons, have shown that alternative exons may respond to membrane depolarization and calcium signaling (reviewed by Sharma and Lou (61)). DM1 models exhibit myotonic discharges, which are involuntary runs of action potentials in muscle fibers (51), resulting in increased calcium signaling (62). To test whether myotonia may contribute to splicing dysregulation, we examined mice with generalized myotonia congenita due to homozygous inactivation of the *Clcn1* chloride channel (*adr* mice, Supplemental Figure S6). While alternative splicing of most transcripts was similar in *adr* and WT quadriceps, seven events differed (Figure 4A). Notably, these myotonia-sensitive splice events were the same group of exons that showed muscle-specific splicing patterns in WT mice. Furthermore, tSNE analysis showed that splicing in *adr* quadriceps clustered away from WT quadriceps and closer to WT muscles having predominant oxidative fiber types, such as soleus and diaphragm (Figure 4b), suggesting that the effects of myotonia on alternative splicing for these events may result from the glycolytic-to-oxidative fiber type conversions that occur in *adr* and HSALR mice (22,63).

### Targeted splice sequencing as a function of *Mbnl* gene dose and CUG^exp^ load in quadriceps

**Figure 5.**
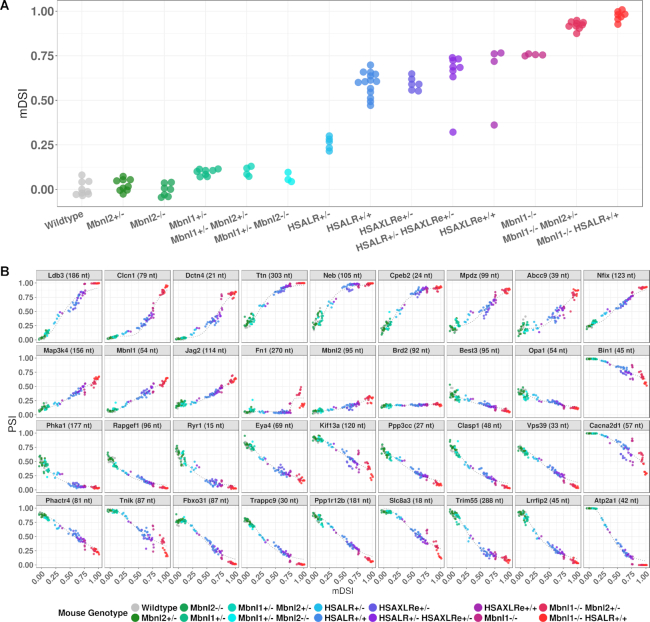
Responsivity of individual splice events and overall splicing index (mDSI) to allelic inactivation of *Mbnl1*/*Mbnl2* or increments of CUG^exp^ expression. (**A**) Based on targeted splice sequencing, mDSI was determined in quadriceps for 97 mice having the indicated genotypes. Note that CUG^exp^ tracts in HSAXLRe mice are twice as long as in HSALR mice. (**B**) PSI responsivity of individual splice events plotted against mDSI, a proxy for residual Mbnl activity. Some events, such as *Neb* or *Phka1*, are more responsive to moderate reduction of Mbnl, whereas others, such as *Clcn1*, are more responsive to changes when CUG^exp^ levels are quite high or Mbnl levels low.

Next, we wanted to determine the effects on splicing of stepwise reductions of Mbnl or increments of CUG^exp^ RNA and develop a summary metric for overall splicing misregulation, the mouse DM splicing index (mDSI). For the latter, we first generated a normalized splicing score for each event, using a linear scale in which the median PSI in WT mice was set at 0 and the 95th percentile for most-affected PSI in DM1 mice was set at 1. We then averaged the normalized splicing scores across all 35 DM1-affected events to generate the mDSI for each sample. Using this metric, we found that heterozygous or homozygous inactivation of *Mbnl2* had no discernable impact on splicing of individual events or mDSI in quadriceps muscle (Figure 5A and Supplemental Figure S7). Heterozygous deletion of *Mbnl1* had a minor effect on splicing of a few events, such as *Ldb3* and *Clasp1*, and a small effect on mDSI, but again there was no additional effect of *Mbnl2* disruption, whether heterozygous or homozygous. Consistent with the minor impact of heterozygous *Mbnl1* deletion on splicing, Mbnl1 protein levels in *Mbnl1*^+/−^ mice were only reduced by 17% (Supplemental Figure S8), confirming reports of post-transcriptional mechanisms for dosage compensation of *Mbnl1* (64,65). In contrast, mice with homozygous deletion of *Mbnl1* showed absence of Mbnl1 protein and major impact on splicing of all individual events and mDSI, and in this case the splicing defects were further aggravated by heterozygous inactivation of *Mbnl2* (Mbnl¾KO mice) and to an even greater extent by concurrent expression of CUG^exp^ RNA (*Mbnl1*^−/−^/HSALR^+/+^ compound homozygotes) (Figure 5A). The latter mice presumably have near-complete loss of Mbnl function in muscle, and they exhibit the most severe splicing misregulation of any mouse model we have examined, accompanied by severe progressive myopathy (X. Lin, W. Wang and C. Thornton, unpublished observations).

We then examined transgenic lines having different levels of CUG^exp^ RNA as a function of transgene expression and repeat length. DMSXL mice carry a human *DMPK* transgene with highly expanded CTG repeats (∼1200 repeats in our colony). However, transgene expression in muscle is low (66,67). Homozygous DMSXL mice showed no effects on individual splice events or mDSI in quadriceps (Supplemental Figure S9), indicating that CUG^exp^ load must exceed a threshold before splicing changes are triggered. In contrast, HSALR mice express shorter repeats (∼220) but at much higher levels. Hemizygous HSALR mice showed a modest increase of mDSI, exceeding that of *Mbnl1*^+/−^ mice (Figure 5A). Homozygous HSALR mice accumulate twice the level of CUG^exp^ RNA as hemizygotes (23) but showed >2-fold higher mDSI, reaching levels slightly below *Mbnl1*^−/−^ mice. Notably, immunoblots showed partial reduction of Mbnl1 protein in homozygous HSALR mice (Supplemental Figure S8), confirming a recent report (68) and suggesting that splicing changes in this model may reflect combined effects of Mbnl1 sequestration plus downregulation. We also examined a newly-derived transgenic line, HSAXLRe, expressing an *ACTA1* transgene similar to HSALR but with twice as many (∼440) CTG repeats. Hemizygous mice in line HSAXLRe showed mDSI comparable to homozygous HSALR mice. However, subsequent efforts to drive CUG^exp^ expression to even higher levels, by breeding HSAXLRe mice to homozygosity or through combinatorial breeding of HSALR and HSAXLRe lines, failed to reach the magnitude of mis-splicing observed in Mbnl¾KO mice, suggesting that capacity of CUG^exp^ RNA from these transgenes to sequester Mbnl has reached saturation.

Using mDSI as a proxy for residual free Mbnl, we plotted the individual PSIs for each splice event as a function of mDSI. In data from the mice with stepwise inactivation of *Mbnl* or increments of CUG^exp^, we observed dose response curves resembling those reported in HEK293 cells having graded doxycycline-inducible expression of MBNL1, or muscle samples from DM1 patients (Figure 5B) (48,49). Similar to the cell and patient data, the splicing responses in mice were described by four-parameter logistic curves (defined by initial PSI, final PSI, EC_50_ and Hill coefficient), which indicated that individual splice events responded differently to marginal reductions of Mbnl. Some events were most responsive to mild reduction (*Cpeb2*, *Clasp1*, *Ldb3*, *Lrrfip2*, *Neb*, *Trim55* and *Ttn*) whereas others were more responsive to moderate or severe reduction (*Abcc9*, *Atp2a1*, *Bin1*, *Cacna2d1*, *Clcn1* and *Jag2*). Both sigmoidal (*Atp2a1* and *Clcn1*) and linear (*Mbnl1*, *Nfix*, *Ppp3cc* and *Vps39*) response curves were observed (Figure 5B). Taken together, these results supported the rationale for using a panel of splice events to monitor Mbnl activity throughout the full range of disease severity.

### Splice events show rapid response to therapeutic reduction of CUG^exp^ RNA

**Figure 6.**
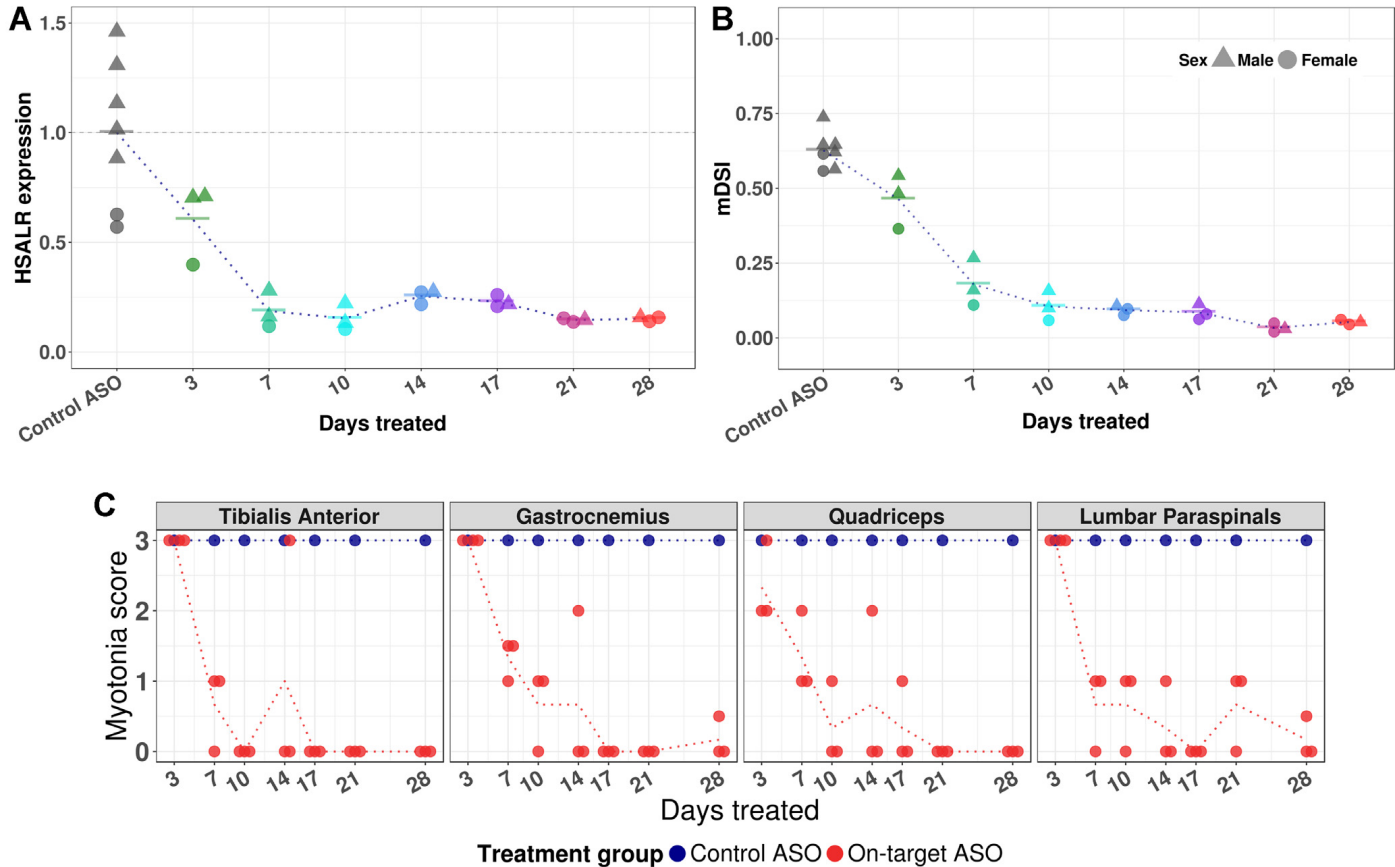
Responsivity of splicing index and myotonia to ASO treatment. (**A**) HSALR mice were treated with an ASO targeting the 3′ UTR of the *ACTA1* transgene mRNA (downstream from the CUG^exp^ tract) or control ASO having no endogenous target. ASOs, conjugated to palmitic acid, were injected subcutaneously at 25 mg/kg twice weekly for up to 4 weeks. Quadriceps was obtained at each time point from three mice treated with active ASO and 1 mouse treated with control ASO. Reduction of transgene mRNA was near-maximal after one week of treatment. Transgene mRNA was determined by RT-qPCR relative to *Gtf2b* and plotted against mean value of control mice, set at 1. (**B**) The response of the composite splicing index, mDSI, is parallel to transgene mRNA knockdown in (A), but slightly delayed. (**C**) Myotonia was assessed by electromyography of indicated muscles in anesthetized mice prior to sacrifice. Myotonic discharges (repetitive action potentials) were scored by blinded examiner on qualitative scale. Myotonic discharges with nearly all needle insertions = 3, >50% = 2, <50% = 1 and 0% = 0. Dotted lines indicate mean transgene expression, mDSI, or myotonia scores in respective treatment groups.

In mice having constitutive CUG^exp^ expression or Mbnl deletion, the results above indicated that splice events reflected differences in the steady state levels of CUG^exp^ or Mbnl accumulation. Next we wanted to assess the dynamic response to therapeutic intervention. For these studies we used RNase H-active antisense oligonucleotides (ASOs) to reduce levels of CUG^exp^ RNA in HSALR mice. Previously we showed that ASOs targeting the *ACTA1* 3′ UTR caused greater than 80% reduction of transgene mRNA in hindlimb muscle when administered by subcutaneous injection of 25 mg/kg twice weekly for 4 weeks (31). In the current study we used the same ASOs and dose regimen, except that ASOs were conjugated to palmitic acid, a modification that improves biodistribution to skeletal muscle (32,69,70). Mice were sacrificed at 3- to 4-day intervals to determine the time course of response. Surprisingly, we found partial reduction of transgene mRNA in quadriceps as early as three days after the first injection, reaching near-maximal reduction (85% knockdown) by day 7, with little additional knockdown from day 7 to 28 (Figure 6A). This apparent ceiling on knockdown efficiency is partly a function of the transgene integration. The integration site was recently mapped to chromosome 2:48,172,446, producing a transgene array in which the final copy is missing the ASO targeting site (Laurent Bogdanik, personal communication). As a result, ∼10% of transgene mRNA is not susceptible to knockdown by this ASO (M. Tanner, C. Thornton, unpublished). Nevertheless, as transgene knockdown occurred, splice sequencing showed a parallel reduction of mDSI, with near-maximal improvement by day 10 and strong correlation between transgene knockdown and splicing rescue (*R*^2^ = 0.86) (Figure 6B). Following rescue of alternative splicing, there was marked reduction or elimination of myotonia in hindlimb and paraspinal muscles (Figure 6C), whereas mice treated with a control non-targeting ASO conjugate showed no effects on transgene expression, mDSI, or myotonia. We also performed a single-injection study using the same ASO conjugate at ascending doses from 12.5 to 100 mg/kg. At 10 days post-injection, we again found parallel reduction of transgene mRNA and mDSI, accompanied by myotonia elimination at higher doses (Supplementary Figure S10). In both experiments, the splicing patterns reverted to WT for 31 of 35 splice events, and for these events the plots of individual PSI versus mDSI during therapeutic rescue suggested that splicing outcomes maintained the same relationship to free Mbnl as in mice with various levels of constitutive CUG^exp^ expression or *Mbnl* loss (Supplementary Figure S11). However, four events—*Cpeb2*, *Neb*, *Phka1* and *Ttn*—deviated from these curves and showed delayed or incomplete response, even after 4 weeks of treatment. These exons were among the subset that showed muscle-specific and myotonia-sensitive splicing, again suggesting that they reflect longer-term tissue remodeling rather than ambient free Mbnl at the time of tissue sampling.

## DISCUSSION

Recently there is growing interest in RNA as a therapeutic target, with DM1, Huntington disease, ALS, transthyretin amyloidosis, and other dominantly-inherited genetic disorders at the forefront (71). Among these, DM1 presents an interesting opportunity for biomarker-driven drug development because the therapeutic targets are well-defined and tissue sampling to obtain muscle RNA is a minor procedure. However, direct determination of CUG^exp^ load is problematic for reasons cited above. Here we have focused on splicing changes as indicators of CUG^exp^ toxicity and therapeutic response because (i) the mechanistic link of splicing dysregulation to CUG^exp^ load and free Mbnl is well established; (ii) the analytical precision for measuring the ratio of two splice products from the same gene can be exceptionally good; (iii) comparison to reference genes is not required, reducing assay and sampling variance and (iv) the analysis is convertible to high-throughput sequencing readouts, which facilitates cross-study comparisons and development of reference standards. Importantly, this approach does not involve any assumption that Mbnl-related splicing defects provide a unitary explanation for clinical features of DM1. Other putative mechanisms, such as CELF1 upregulation, activation of signaling cascades, or expression of repeat-associated non-AUG-dependent translation products, may certainly contribute (72–75). However, the central pathogenic factor for all of these mechanisms is CUG^exp^ RNA, whose accumulation creates a finite capacity to titrate Mbnl, which in turn is reflected by outcomes for Mbnl-dependent splice events. As such, targeted splice sequencing provides evidence for the on-target mechanism of agents that reduce the synthesis or accelerate the degradation of CUG^exp^ RNA, increase MBNL protein, or inhibit MBNL-CUG^exp^ binding.

In the current study, we found extensive changes of the muscle transcriptome in mouse models of DM1 and concordant effects of CUG^exp^ expression or *Mbnl* deletion. Although previous work has suggested that other RBPs, including CELF1, HNRNP H, and RBFOX, may contribute to splicing misregulation in DM1 (73,76–79), our results indicated that Mbnl loss may account for nearly all effects on alternative splicing in the HSALR model and most of the effects on gene expression, in line with previous studies using RT-PCR or microarrays (8,36,40). We also found that variants of the YGCY core motif for Mbnl binding were the strongest predictors of splicing misregulation in DM1 models (35,49), again suggesting that many of the affected exons were directly regulated by Mbnl proteins. In fact, the only non-Mbnl motif that predicted splicing misregulation was CUAAU, a consensus binding site for Quaking (Qk), another factor that regulates alternative splicing during muscle development (39). Further studies are needed to examine the potential role of Qk in DM1. While we did not observe changes of *Qk* splicing or expression by RNAseq, a previous study reported that Mbnl1 may bind to the 3′ UTR of *Qk* mRNA (37), potentially affecting localization or translation. Alternatively, it is possible that Mbnl loss triggers downstream events, such as myotonia, that have post-translational effects on Qk function. It also remains possible that other splicing factors are affected by Mbnl loss but were overlooked in our motif analysis because the cognate binding sequences are poorly conserved, degenerate, or structure-dependent.

Muscle fibers are classified into distinct types according to their metabolic and contractile properties (reviewed by Schiaffino and Reggiani (80)). While the distribution of fiber types varies among muscles, these properties are not fixed. Repetitive muscle activity, whether by exercise, electrical stimulation, or myotonia, can trigger transcriptional reprogramming and conversion from glycolytic to oxidative fiber types (62,63,81). For example, HSALR, *Mbnl1*^−/−^ and *adr* mice all exhibit myotonia and increased frequency of oxidative fibers (16,22,63), and we previously reported that 24% of changes in gene expression in HSALR mice are likely due to myotonia-dependent transcriptional reprogramming, as evidenced by parallel changes in *adr* mice (40). In contrast, the current study is the first to show that myotonia also triggers changes of RNA processing. Twenty percent of splicing changes on our panel were affected similarly in *adr* as DM1 mice. Interestingly, these same events were differentially regulated in WT muscles having predominance of fast-twitch glycolytic (gastrocnemius and quadriceps) *vs* oxidative fiber types (masseter, diaphragm, and soleus), suggesting that fiber type conversions are the fundamental driver of these particular splicing changes. The factors responsible for this process are presently unknown, but Mbnl does not appear to be directly involved since the other 80% of events on our panel showed neither muscle-specificity or myotonia sensitivity, including several exons that are proven direct targets of Mbnl.

In developing therapy for chronic diseases, the timing and extent of clinical response may depend on factors other than drug activity, such as cell plasticity and regenerative potential. It is possible that target engagement, even if highly successful, may simply stabilize symptoms or slow progression. Amid this uncertainty about when and how a clinical benefit will manifest, evidence that a drug has reached its target and had the intended effect can be extremely useful, but how early and reliably can this actually be determined? In this regard, the nuclear RNA foci in DM1 are dynamic structures that form through assembly of ribonucleoprotein complexes and liquid-liquid phase separation (82–86). Although segregated into foci, the mutant *DMPK* transcripts remain metabolically active and exhibit turnover rates comparable to WT counterparts (3,87). In other well-studied examples, site-specific endonucleolytic cleavage of mRNA, whether by ASO/RNase H or siRNA-ago2, was followed by rapid exonucleolytic clearance of the cleavage fragments (88). Similarly, we have found in mouse models that clearance of CUG^exp^ RNA was highly effective after ASO/RNase H cleavage, regardless of whether cleavage occurred up- or down-stream of the repeat tract (23,31). Accordingly, we expected that target cleavage in HSALR mice would produce rapid release of Mbnl, and that any lag between target knockdown and splicing rescue would primarily reflect the turnover kinetics of Mbnl-dependent transcripts. Since half-lives of most transcripts are in the range of 2–6 h (89,90), we expected minimal lag between target knockdown and splicing recovery. The data from our ASO studies agreed with this prediction, except in the case of a few events that seem to reflect longer-term disease adaptations, perhaps related to fiber type reprogramming.

The clinical response to therapy, however, will inevitably lag behind. Correction of the transcriptome will be followed by recovery of the proteome, but in muscle this is bound to be slow. Bulk protein turnover rates in muscle are only 1.1% per day *in vivo* in healthy individuals and slower in DM1 patients than in healthy controls (91,92). Furthermore, beyond the recovery of the proteome, the reversal of muscle atrophy and normalization of myofiber architecture is likely to depend on other factors, such as exercise, nutrition, fibrosis, and regenerative capacity, making it hard to predict the time course of response and potential for recovery. However, the effects on myotonia may be a special case, due to the unique circumstances of the chloride channelopathy. The mis-spliced *Clcn1* mRNA is frame shifted, producing an unstable mRNA that is subject to nonsense-mediated decay (25), and encoding a truncated protein that is missing domains required for channel dimerization or ion conductance (93,94). Accordingly, the rescue of chloride conductance may depend exclusively on synthesis of new channels rather than turnover of preexisting mis-spliced isoforms. Since correction of myotonia is believed to occur when chloride conductance rises to only 30% of WT levels (95), and *Clcn1* splicing was partially corrected as early as 3 days after ASO injection, it is not surprising that we observed partial reduction of myotonia as early as day 7 of ASO treatment and complete resolution by day 10 at higher doses. These results support the idea that myotonia may provide a rapid-response physiological indicator of Mbnl release, which is closely linked to correction of splicing defects.

## DATA AVAILABILITY

RNAseq data for WT, HSALR and Mbnl¾KO mice are available in the NCBI Sequence Read Archive at accession PRJNA625451. Isoform-specific counts and PSIs for all samples used for targeted RNAseq are provided in Supplemental Data Tables S4 and S5, respectively.

## Supplementary Material

gkab022_Supplemental_Files
