# Supplementary material for: Targeted splice sequencing reveals RNA toxicity and therapeutic response in myotonic dystrophy
Source: Nucleic Acids Res. 2021 Jan 27;49(4):2240–54. doi: 10.1093/nar/gkab022 (PMC7913682; doi:10.1093/nar/gkab022)
Supplement: gkab022_Supplemental_Files [file gkab022_supplemental_files.zip › Targeted splice sequencing reveals RNA toxicity and therapeutic response in myotonic dystrophy (Supplement) (Revised).docx]

**a**


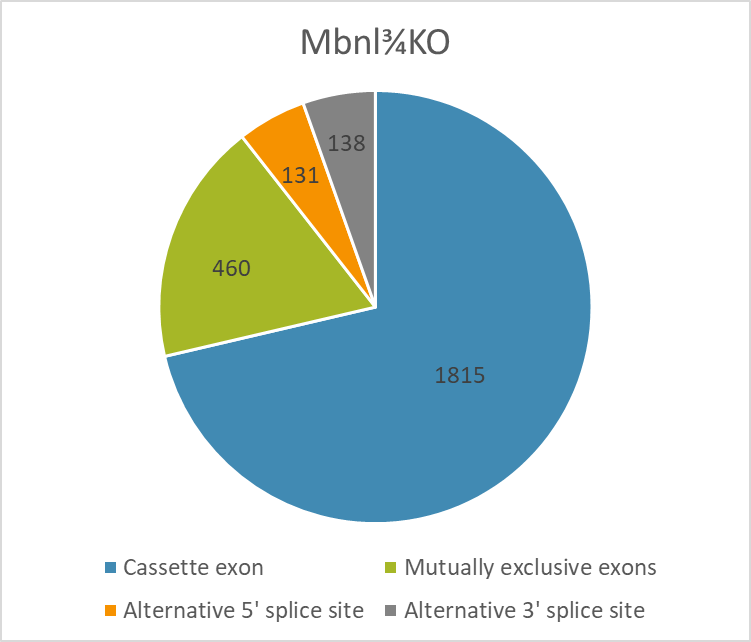

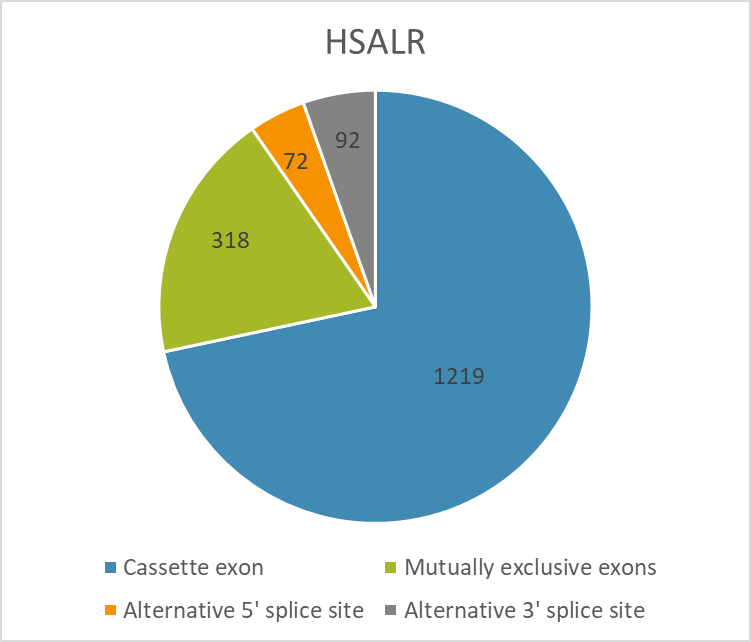


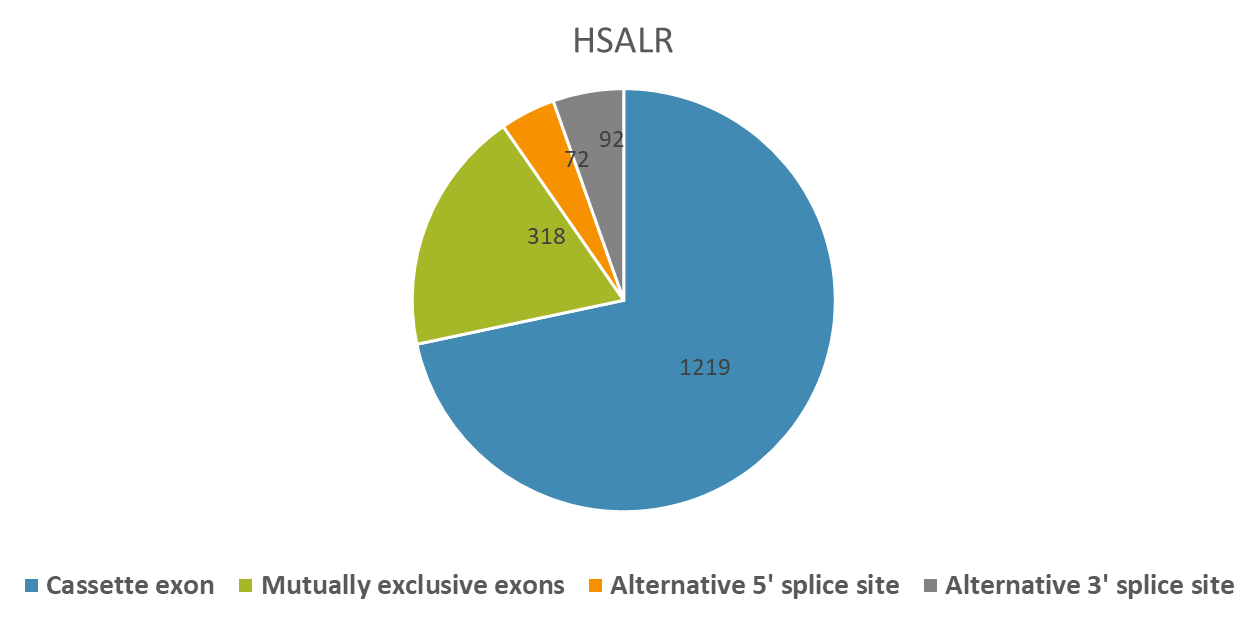


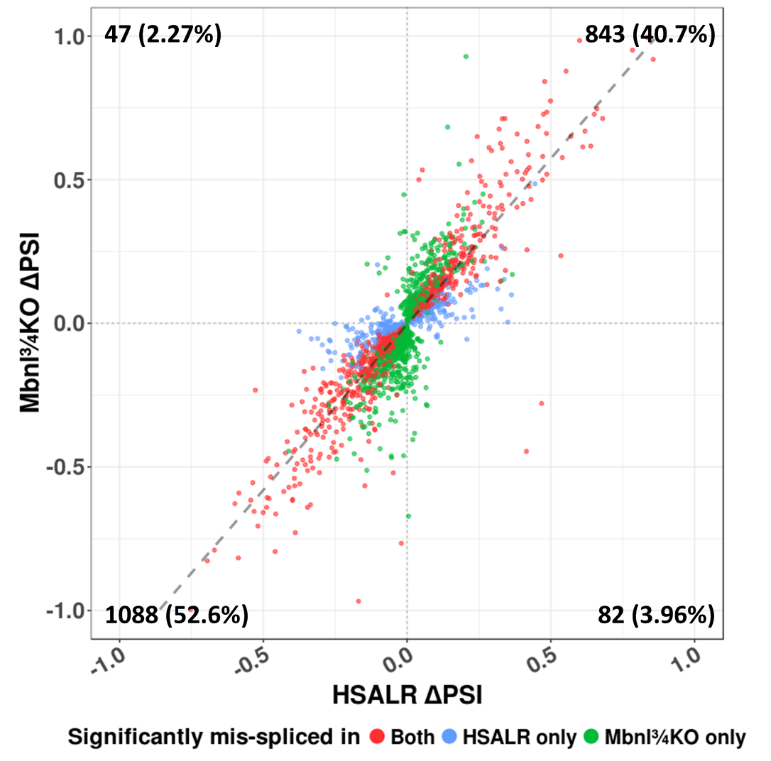

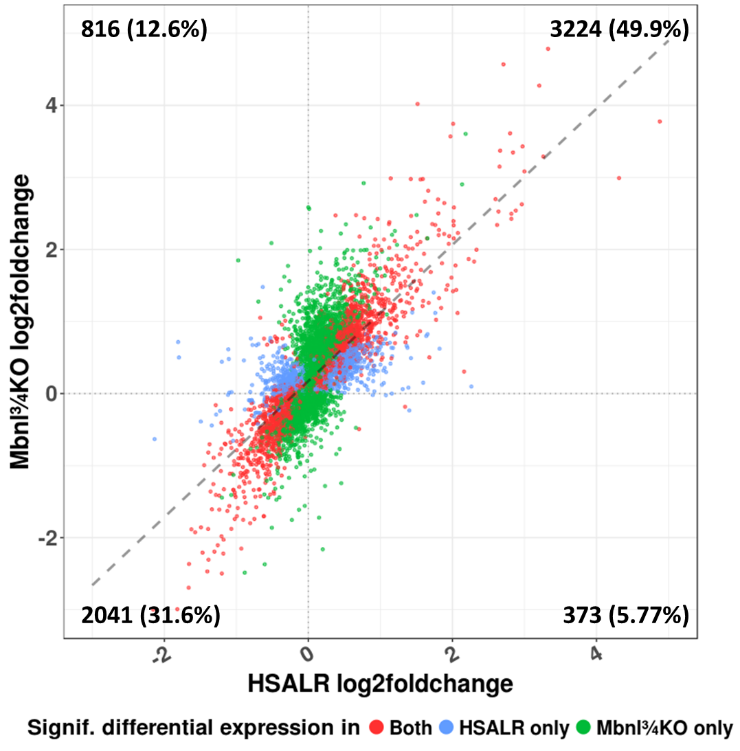


**b**

**c**

**Supplemental Figure 1. Concordance of splicing misregulation and differential gene expression in quadriceps of HSALR and Mbnl¾KO mice, as compared to WT controls.** ***a***, the most common abnormality of alternative splicing in HSALR and Mbnl¾KO quadriceps (adj. *p* < 0.05, no threshold for effect size) involved differences of inclusion or skipping of alternative cassette exons. ***b***, of 2,070 alternative cassette exons that were significantly misregulated in HSALR or Mbnl¾KO mice, 93% exhibited concordant changes in both models. The number of splice events in each quadrant is indicated. Splicing changes in the two models were highly correlated (*R*^2^ = 0.75) but tended to be greater in magnitude in Mbnl¾KO mice (slope of regression line = 1.15). ***c***, among 6,463 genes that were differentially expressed in either HSALR or Mbnl¾KO quadriceps (adj. *p* < 0.05, no threshold for effect size), 81% showed concordant changes of gene expression (*R*^2^ = 0.54, slope = 0.95). The number of differentially expressed genes in each quadrant is indicated.


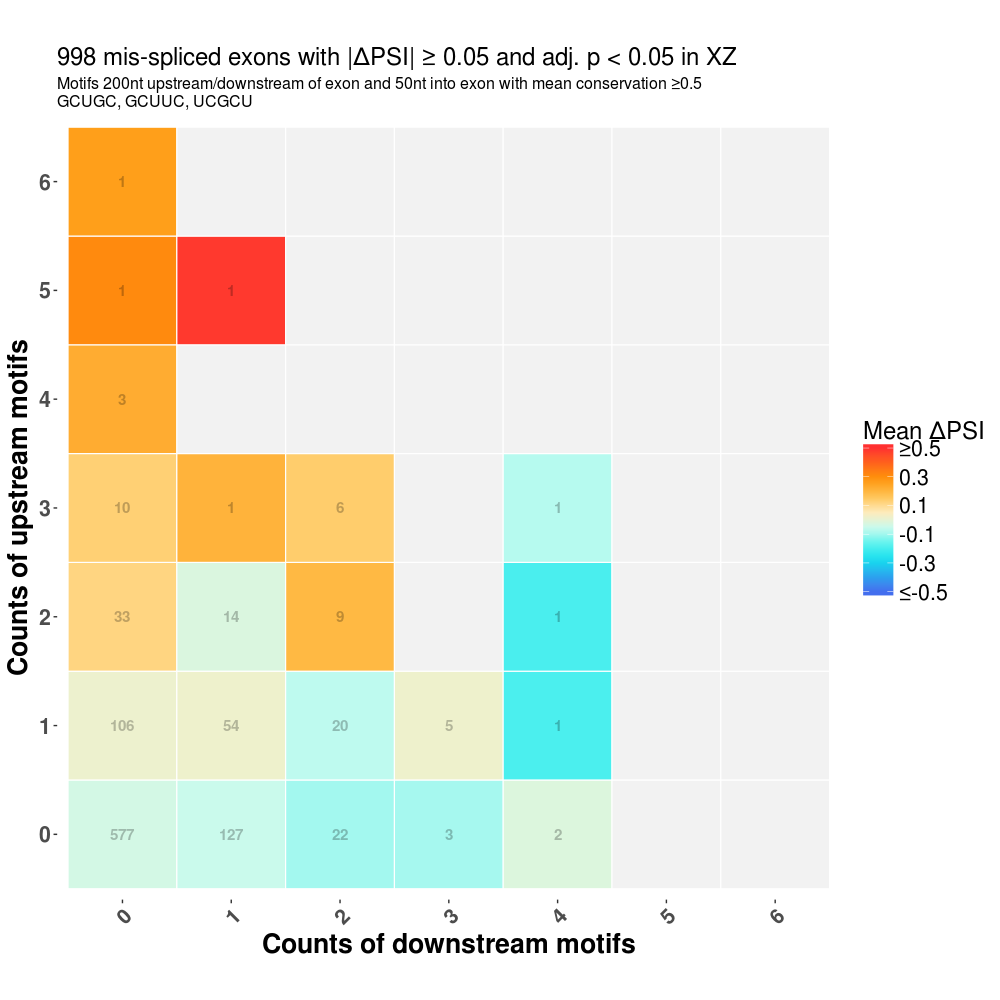


**a**

**HSALR (GCUGC, GCUUC, and UCGCU)**

**b**

**Mbnl¾KO (GCUGC, UCUGC, and UGGCU)**


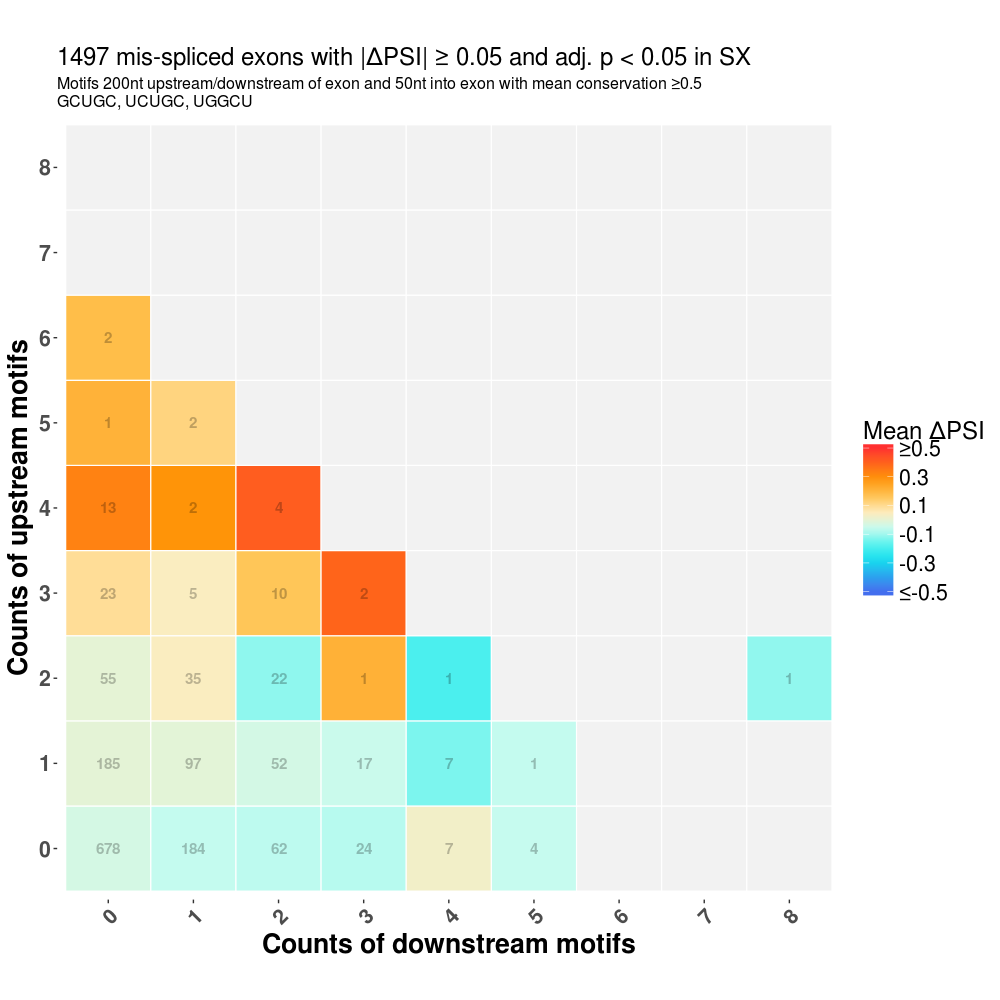

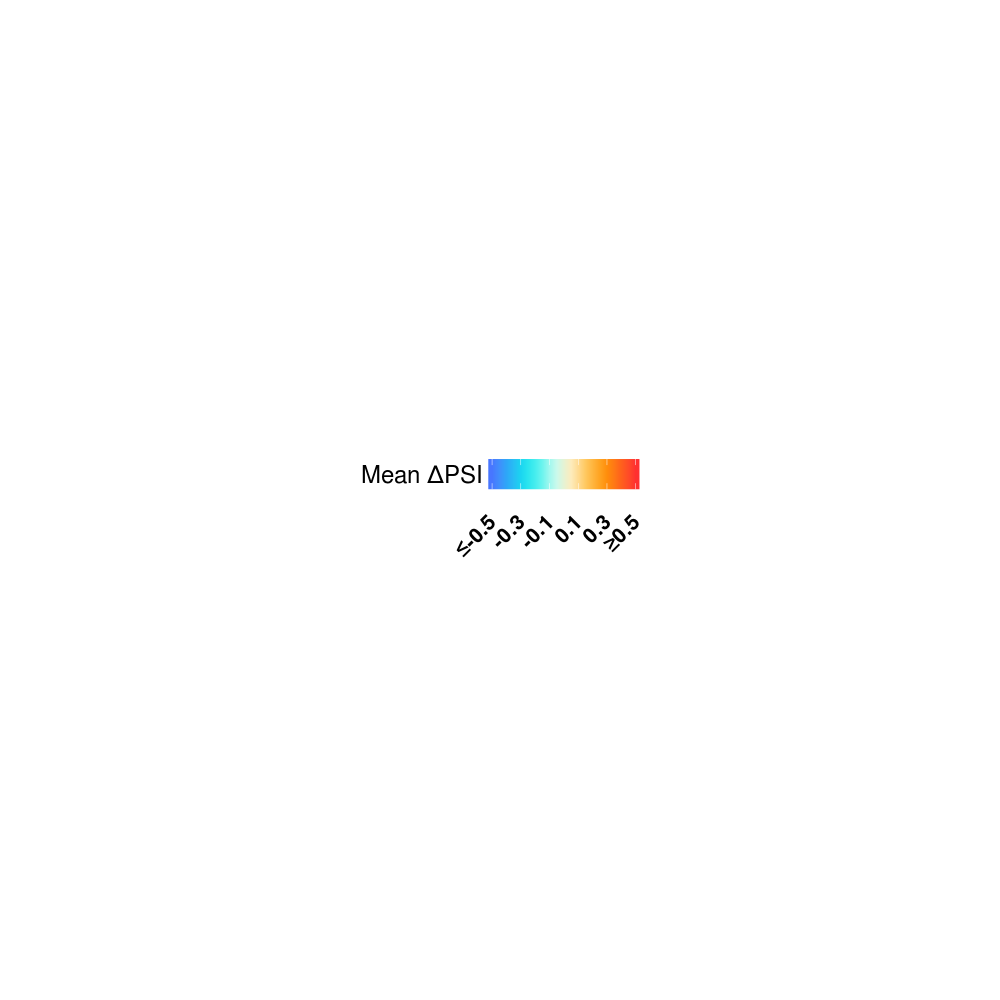

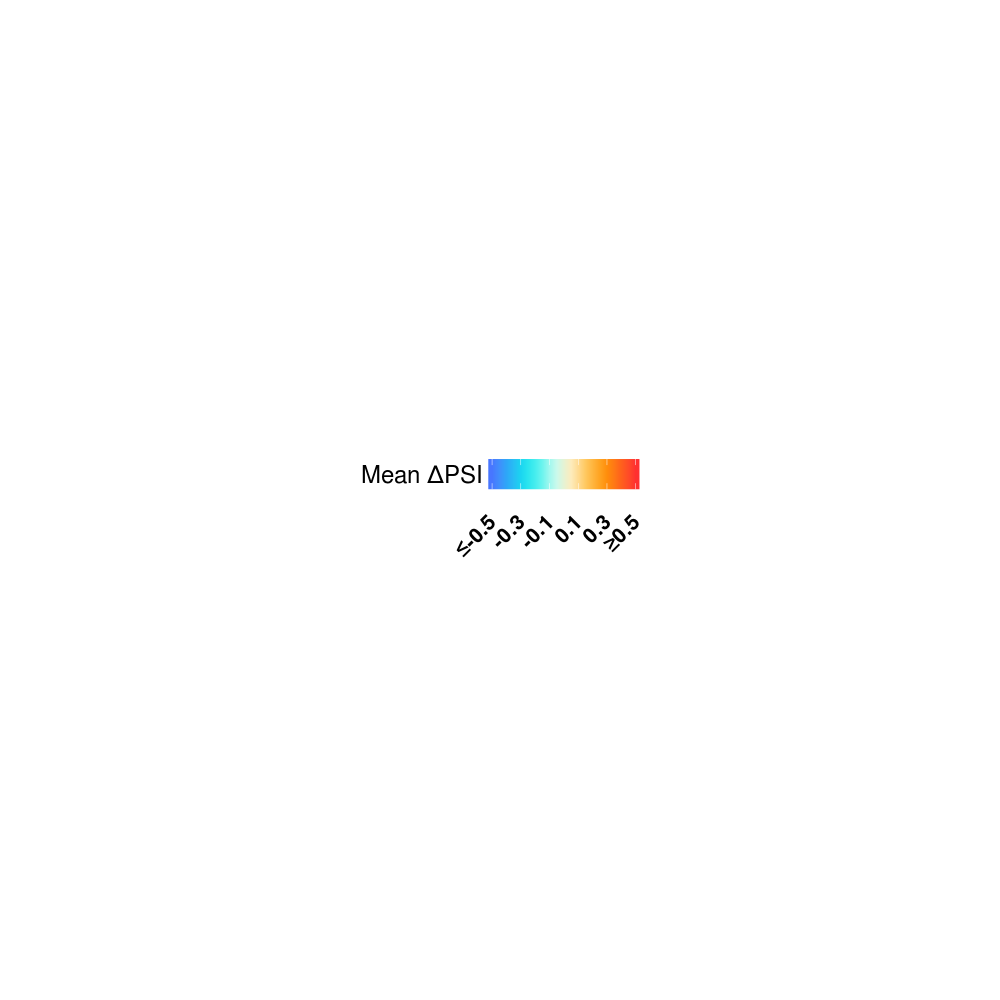


**Supplemental Figure 2**. **Multiplicity and position of sequence motifs is associated with exon inclusion or skipping in DM1 models.** The three pentamers having strongest association with splicing misregulation (Supplemental Tables 1 and 2) are listed for HSALR (**a**) and Mbnl¾KO (**b**) mice, each of which constitutes a potential Mbnl binding site. For each mis-spliced exon, these motifs were counted in the 5′ and 3′ flanking sequences (200nt of intron and 50nt of adjacent exon). Each tile depicts exons having the specified number of up or downstream motifs. Within each tile, the mean ΔPSI of corresponding exons is shown by heat map, and the number of exons having the specified motif counts is indicated. For both DM1 models, multiplicity of upstream Mbnl-binding motifs was associated with greater exon inclusion, whereas multiplicity of downstream occurrences was associated with greater exon skipping. Analysis was performed on all misregulated exons with $\left| \Delta PSI \right|\geq0.05$ and FDR-adjusted *p* < 0.05 (1,005 exons for HSALR and 1,506 exons for Mbnl¾KO).

**a**


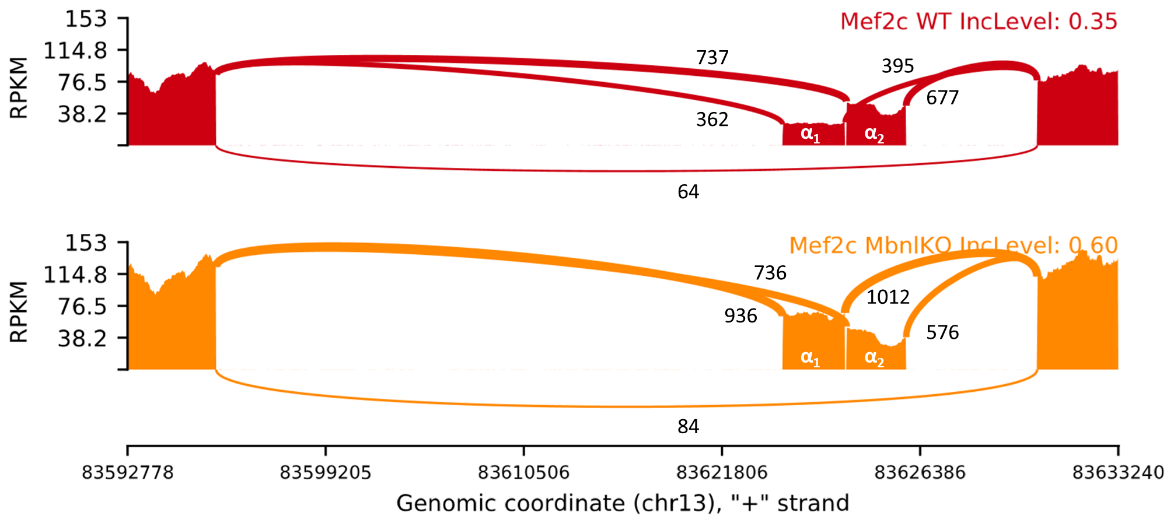

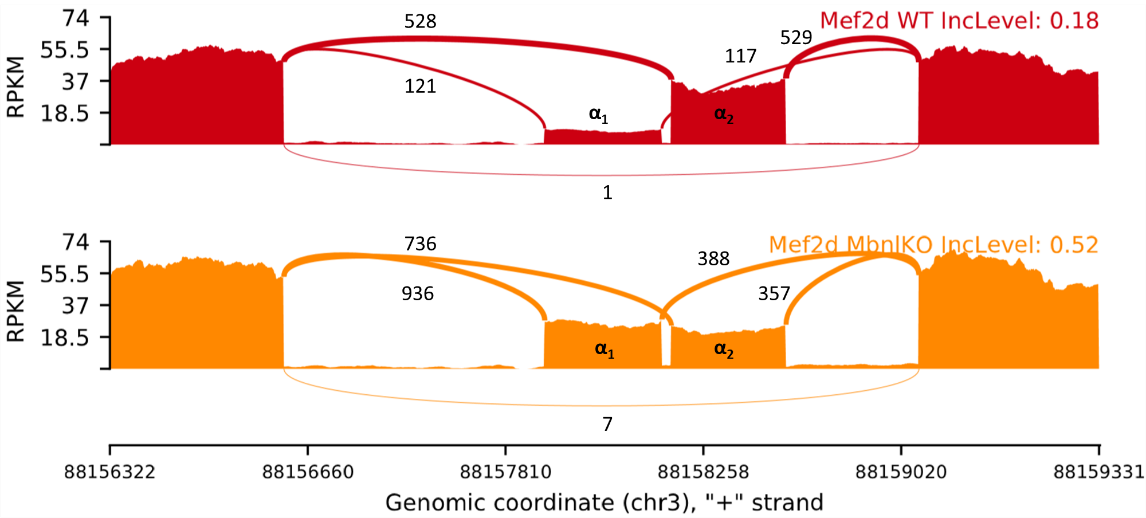


**b**


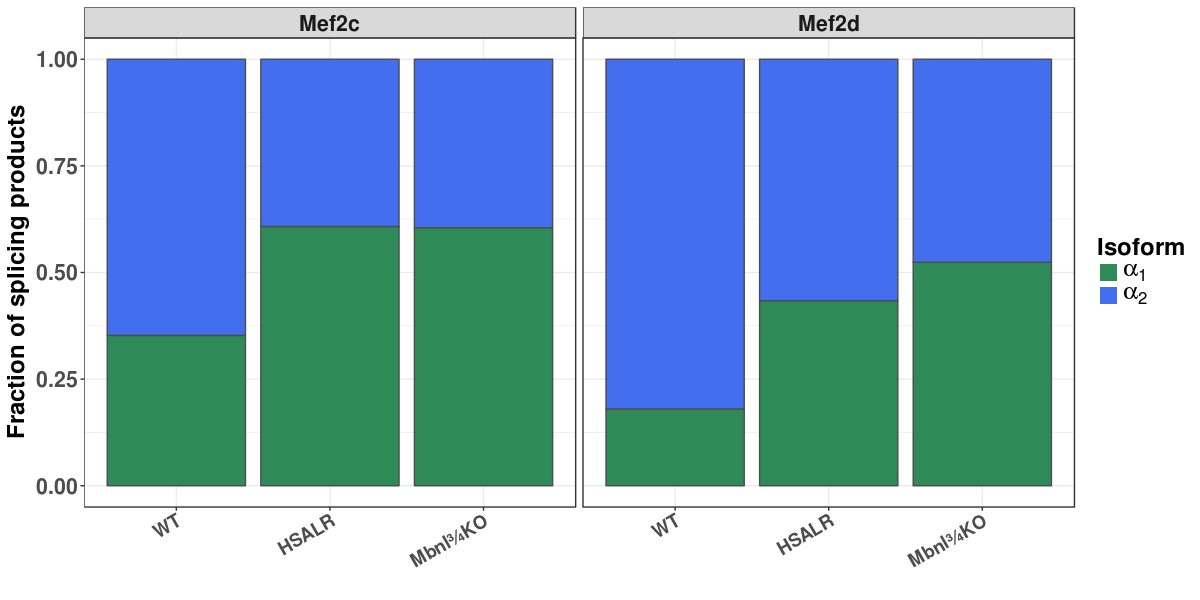


**Supplemental Figure 3**. **Misregulated alternative splicing of *Mef2c* and *Mef2d* in DM1 models**. ***a***, *Mef2c* and *Mef2d* exhibit mutually exclusive alternative splicing of the α_1_ and α_2_ exons, producing isoforms with antagonistic effects on myogenesis and muscle gene expression. Sashimi plots of RNAseq data for *Mef2c* (left) and *Mef2d* (right) showed that Mbnl¾KO mice (orange) exhibit partial reversion to the non-myogenic α_1_ isoforms as compared to WT mice (red). The number of splice junction-spanning RNAseq reads is indicated (cumulative counts for 4 Mbnl¾KO and 4 WT mice). For *Mef2c*, α_1_ exon inclusion ranged from 0.32-0.39, 0.57-0.66, and 0.57-0.65 in WT, HSALR, and Mbnl¾KO mice, respectively. For *Mef2d,* α_1_ inclusion ranges from 0.16-0.19, 0.41-0.49, and 0.48-0.56, respectively. ***b***, HSALR and Mbnl¾KO quadriceps both show an increase of α_1_ isoforms of *Mef2c* (left) and *Mef2d* (right). RPKM, reads per kilobase per million total mapped reads.


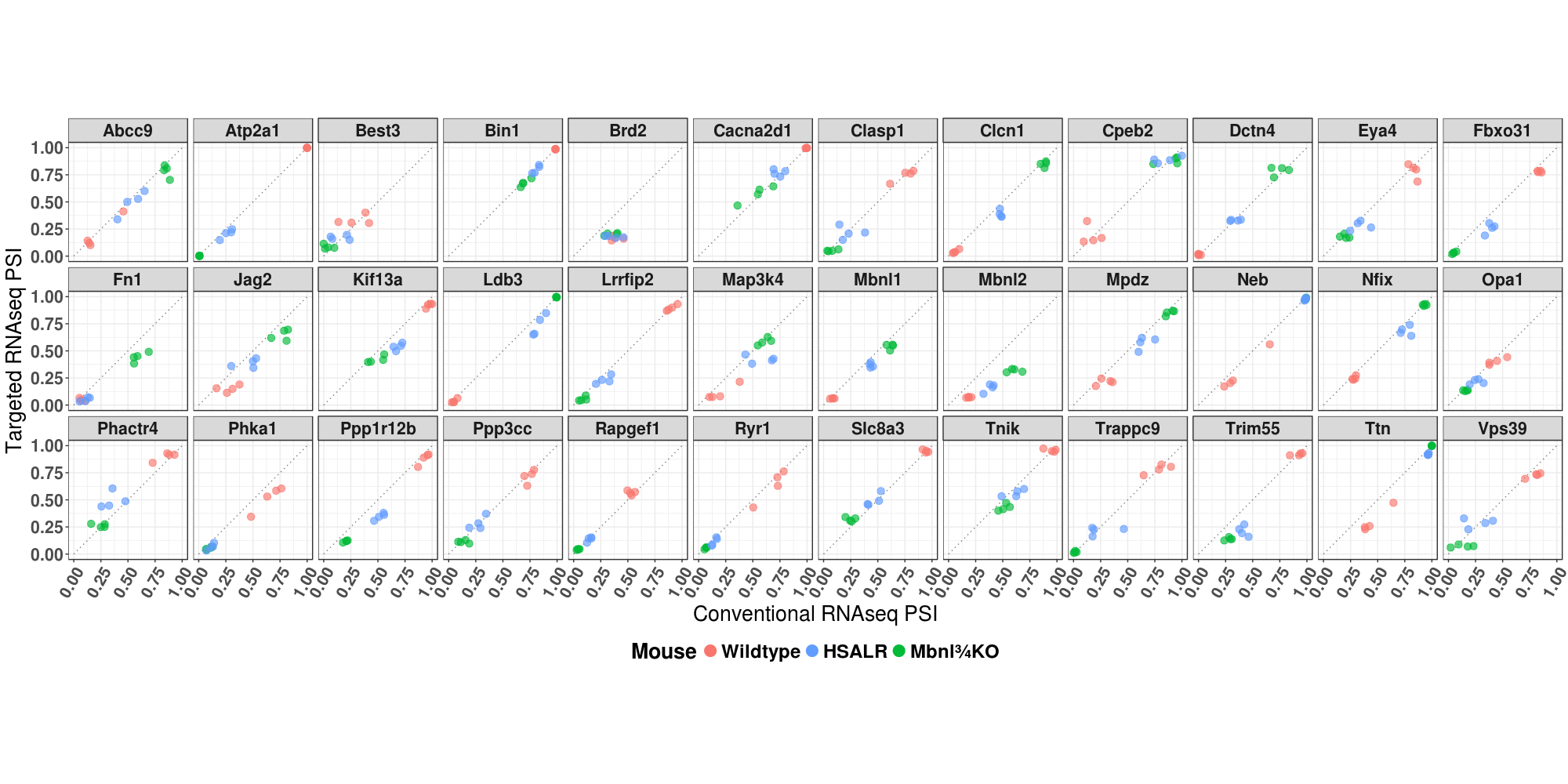


**a**

**b**


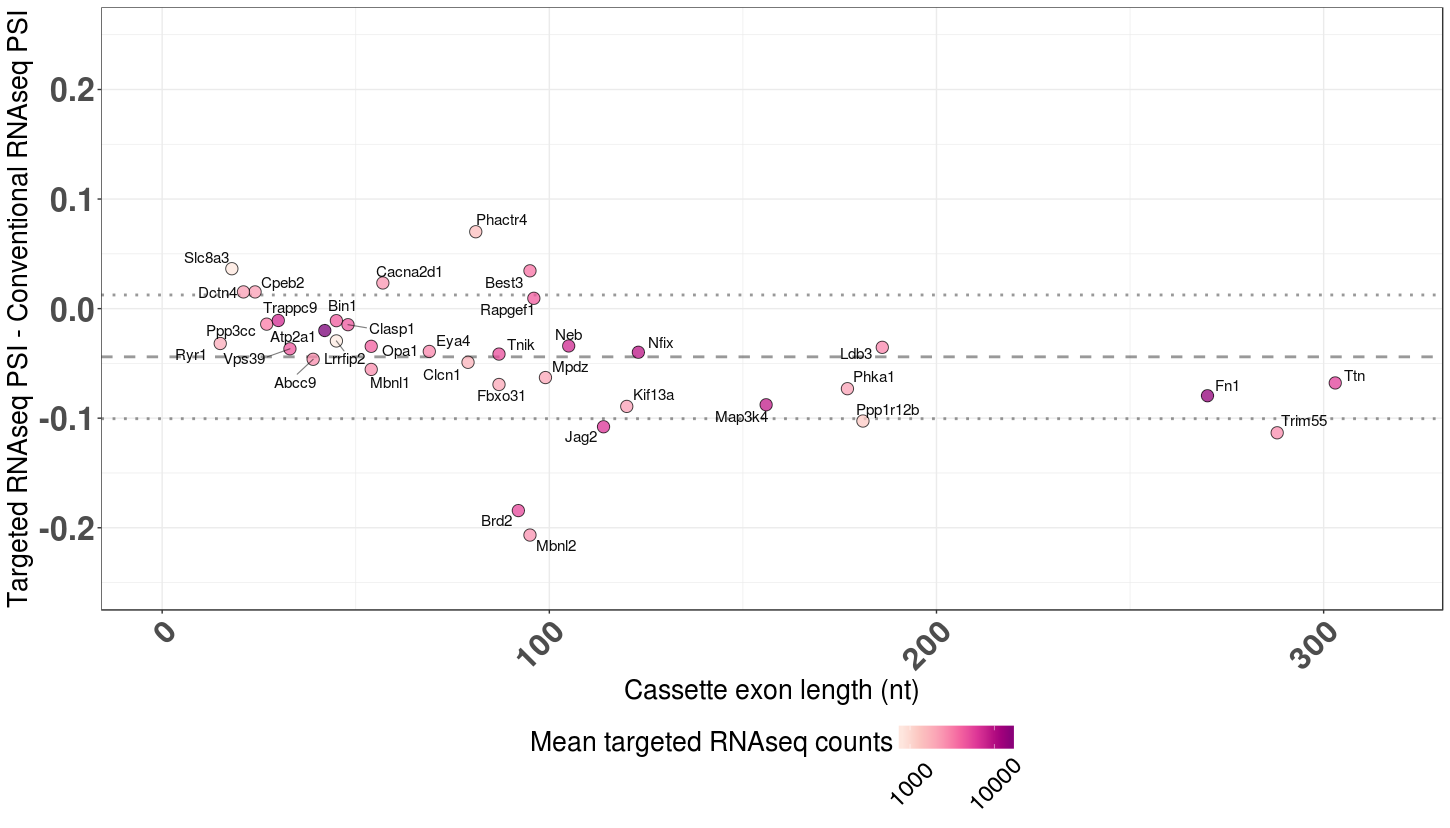

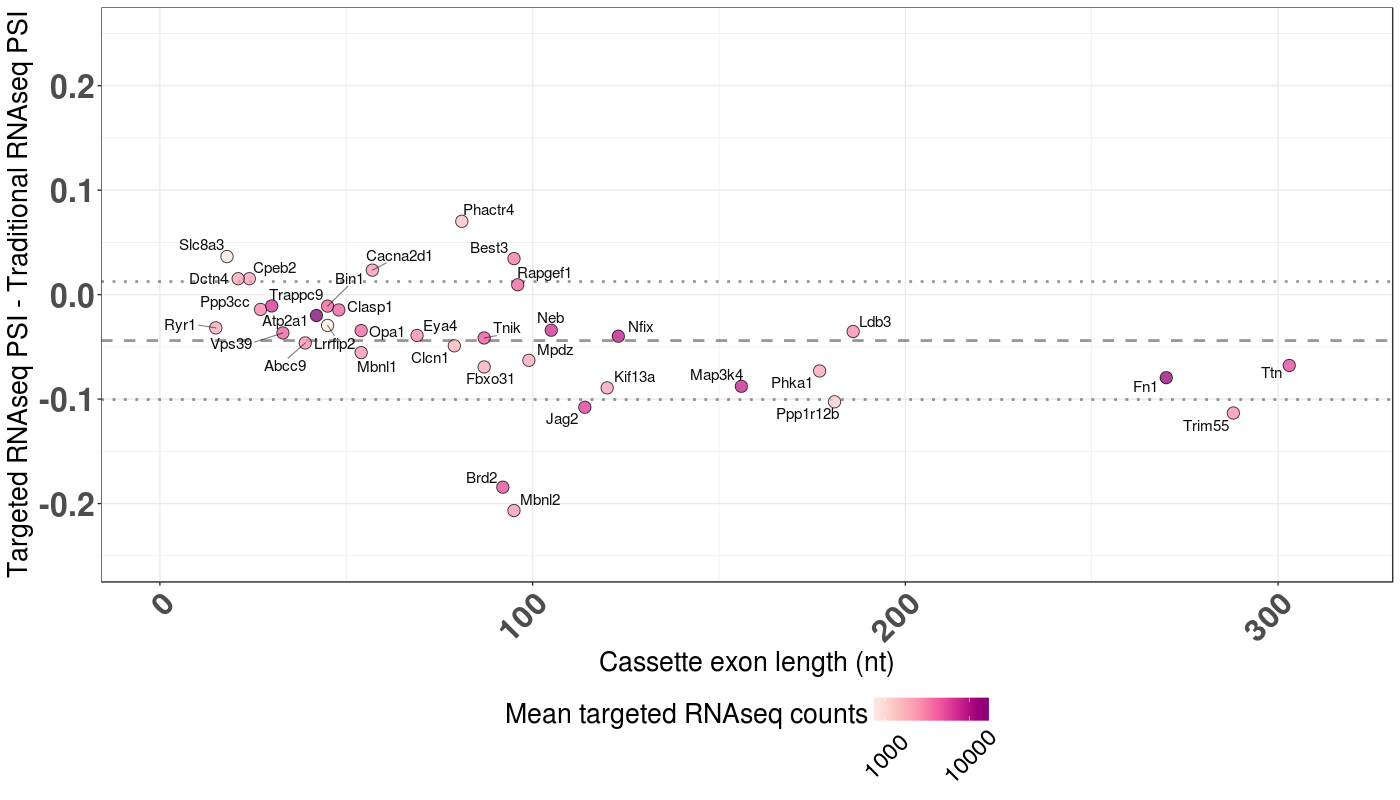


**Supplemental Figure 4.** **Targeted RNAseq underestimates exon inclusion isoforms for some transcripts.** Quadriceps RNA was used for conventional and targeted RNAseq in 12 mice (4 each for HSALR, Mbnl¾KO, and WT mice). PSIs were derived for the panel of 36 splice events using the same isoform counting method for both datasets. ***a***, comparison of PSI quantification by targeted and non-targeted RNAseq across all mice and all splice events. ***b***, overall, the targeted RNAseq exhibited small but significant bias for exon exclusion isoforms (average difference in PSI = -0.04, *p* = 0.00004 by *t* test), that depended partly on length of the alternative exon (*R*^2^ = 0.19, *p* = 0.007 by F-test). Dashed line represents mean difference and dotted lines represent mean ± one standard deviation. *Brd2* and *Mbnl2* were outliers. By conventional RNAseq, both of these events showed partial retention of the upstream intron when the alternative exon was included. The intron-retention isoforms were detected by conventional RNAseq but not by targeted RNAseq, contributing to under-detection of exon inclusion for these events. Note that variance between the two methods is greatest for low abundance transcripts, when conventional RNAseq generated fewer than 120 isoform-specific reads per sample (*Best3*, mean isoform specific reads per sample = 16; *Clasp1*, 71; *Cpeb2*, 24; *Eya4*, 45; *Jag2*, 81; *Map3k4*, 115; *Phactr4*, 35; *Trappc9*, 66; *Vps39*, 50). Isoform specific reads generated for all transcripts by both methods are shown in columns T and U of Supplemental Data Table 3.

**a**

**b**


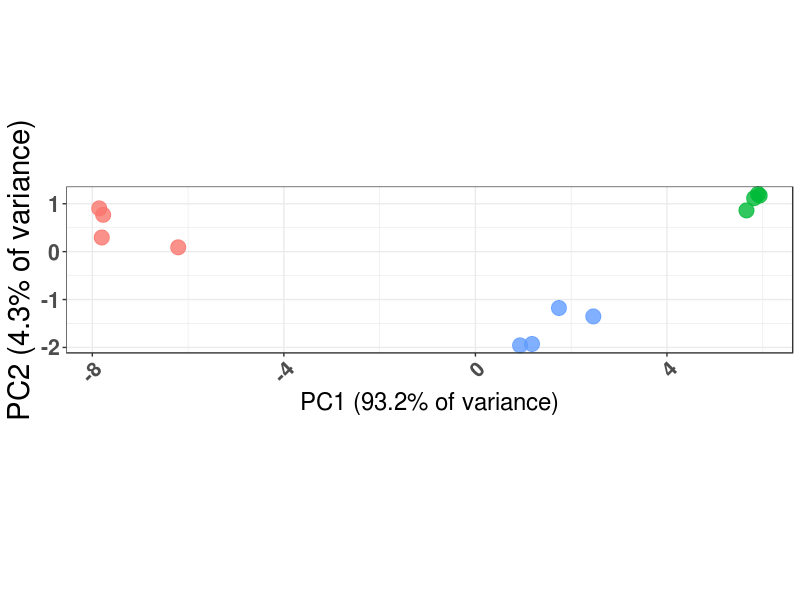

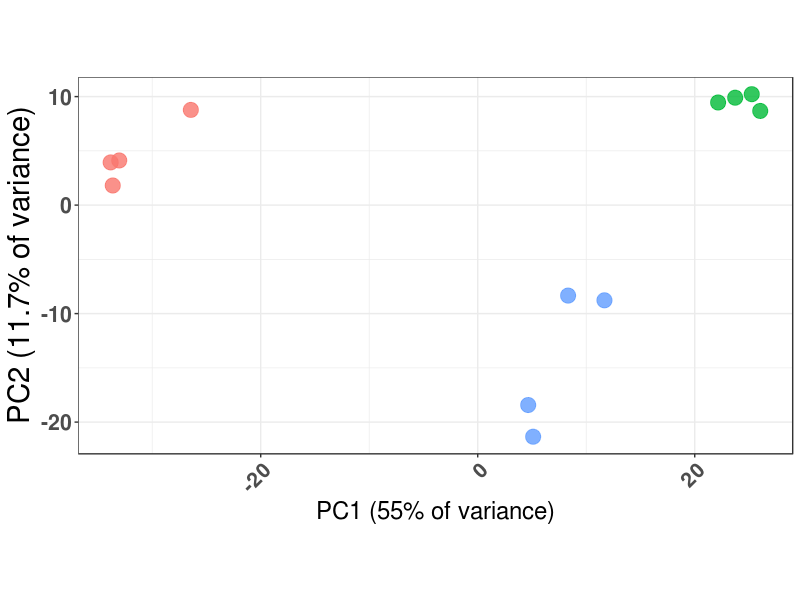


**c**


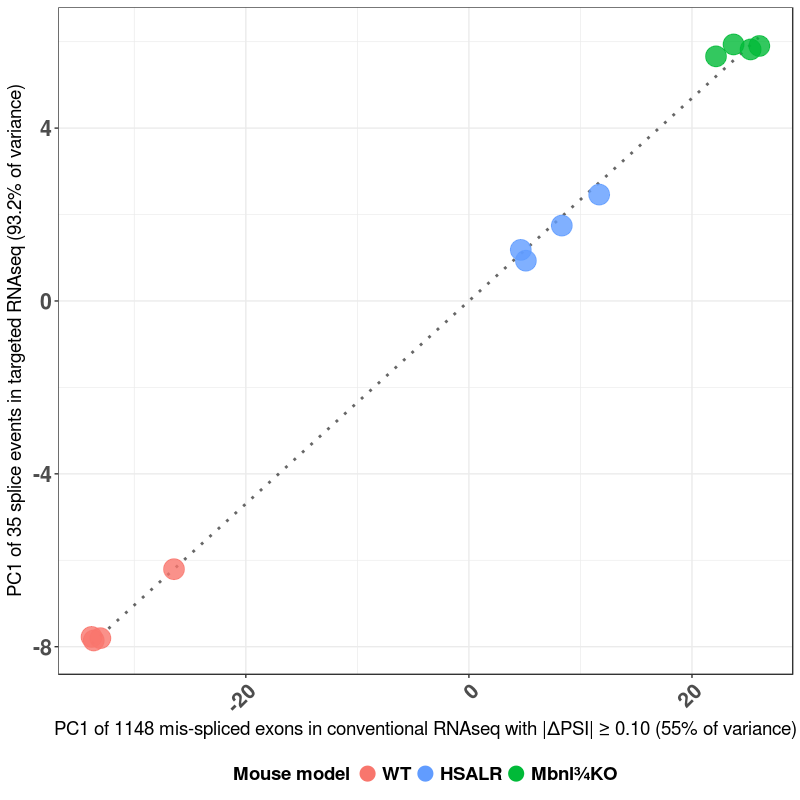

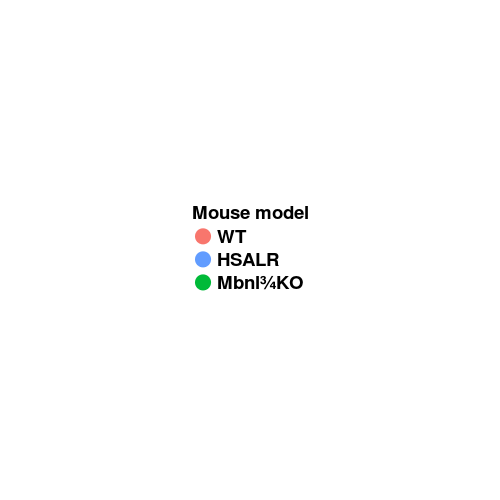


**Supplemental Figure 5**. **A subset of mis-spliced exons recapitulates a large portion of transcriptome-wide splicing changes**. ***a***, principal component analysis (PCA) was performed on conventional RNAseq data using PSIs of 1,148 exons that are mis-spliced (|ΔPSI|>0.10 and FDR-adjusted *p* < 0.05) in either HSALR or Mbnl¾KO mice, producing a first principal component (PC1) that explains 55% of variance in splicing of these exons. ***b***, PCA was also performed on PSIs from targeted RNAseq of the 35 selected DM1-sensitive exons, producing a PC1 explaining 93.2% of splicing variance in these 35 exons. ***c***, both PC1s are strongly associated with each other (*R*^2^ = 0.998).


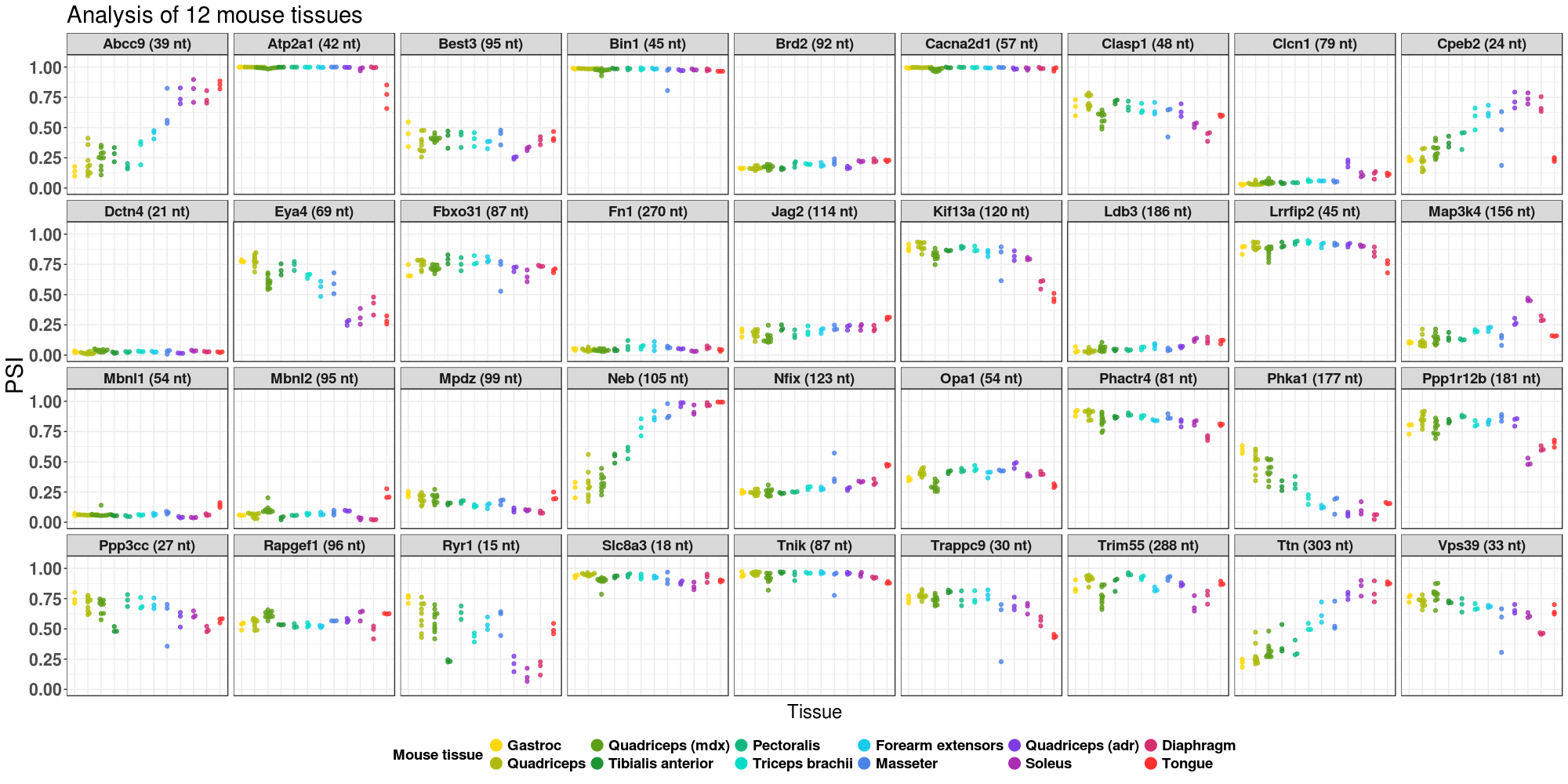

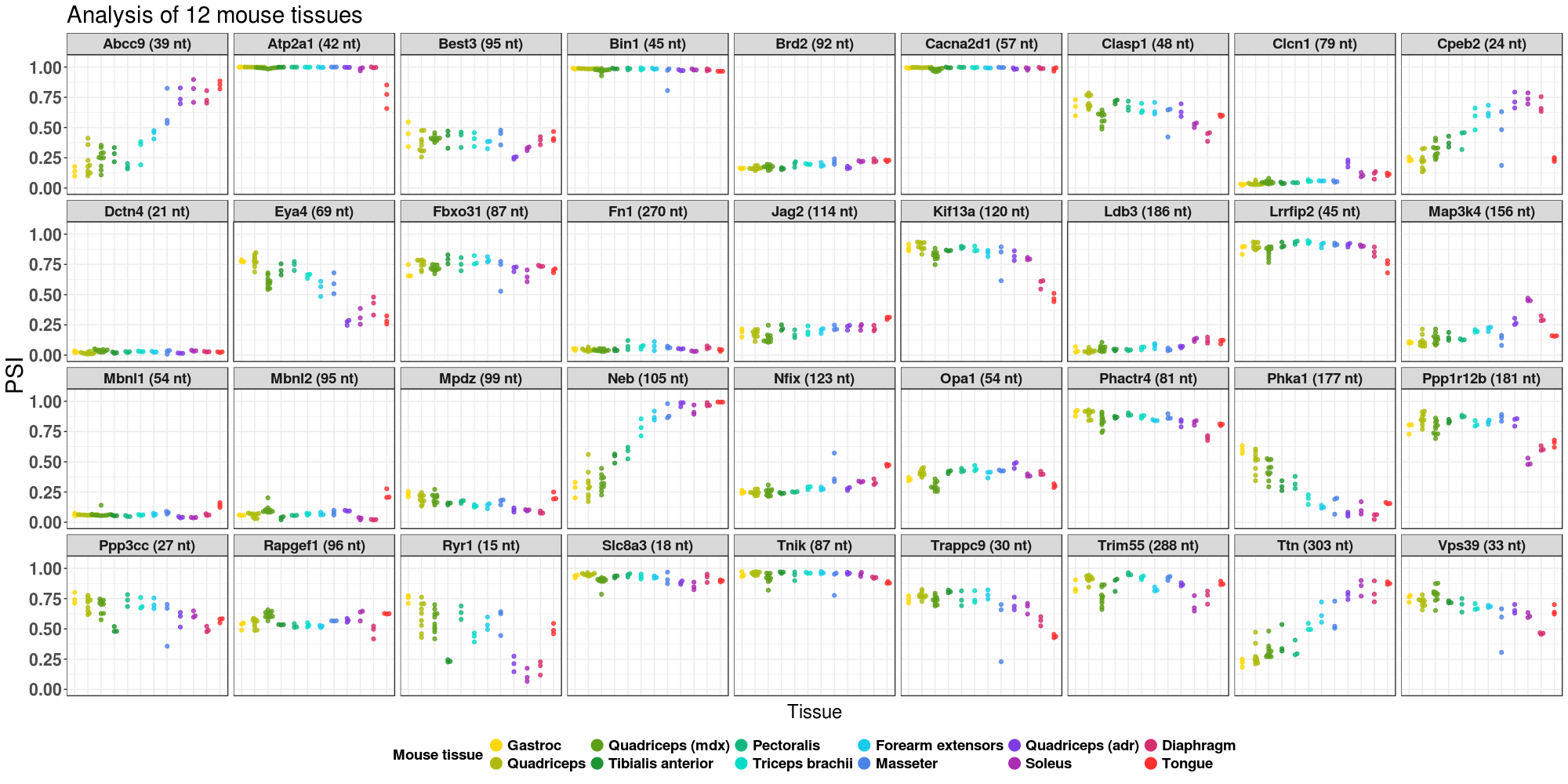


**Supplemental Figure 6**. **Targeted RNAseq of multiple muscles from WT mice and quadriceps from *mdx* or *adr* mice.** Among 35 DM1-affected splice events, most showed similar splicing regulation among WT muscles, but *Abcc9*, *Cpeb2*, *Eya4*, *Neb, Phka1*, *Ryr1*, and *Ttn* did not. Splicing patterns in *mdx* quadriceps, a model for Duchenne muscular dystrophy, did not differ substantially from wildtype quadriceps, whereas *adr* quadriceps differed from wildtype quadriceps and instead appeared similar to wildtype soleus and diaphragm.


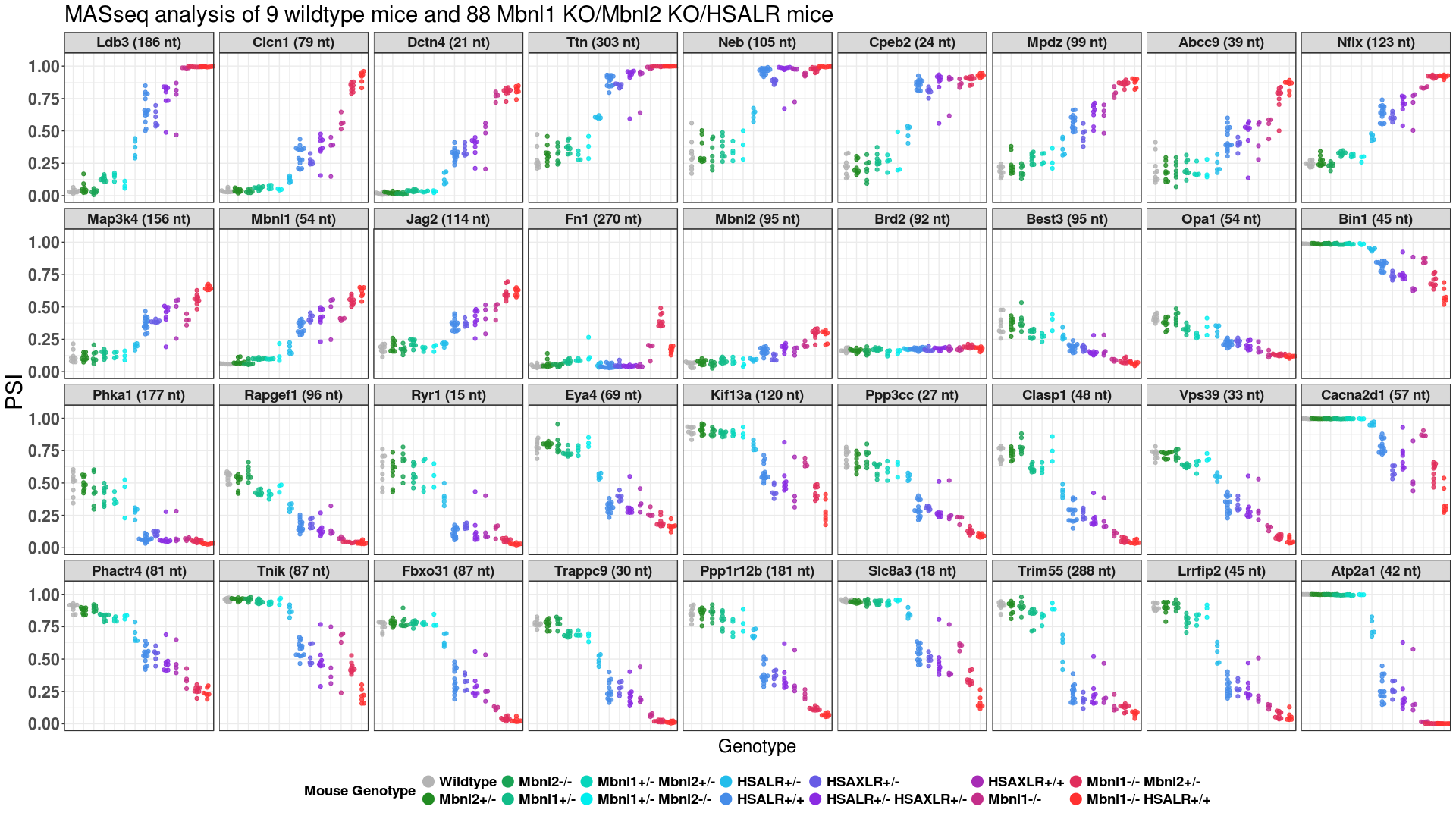

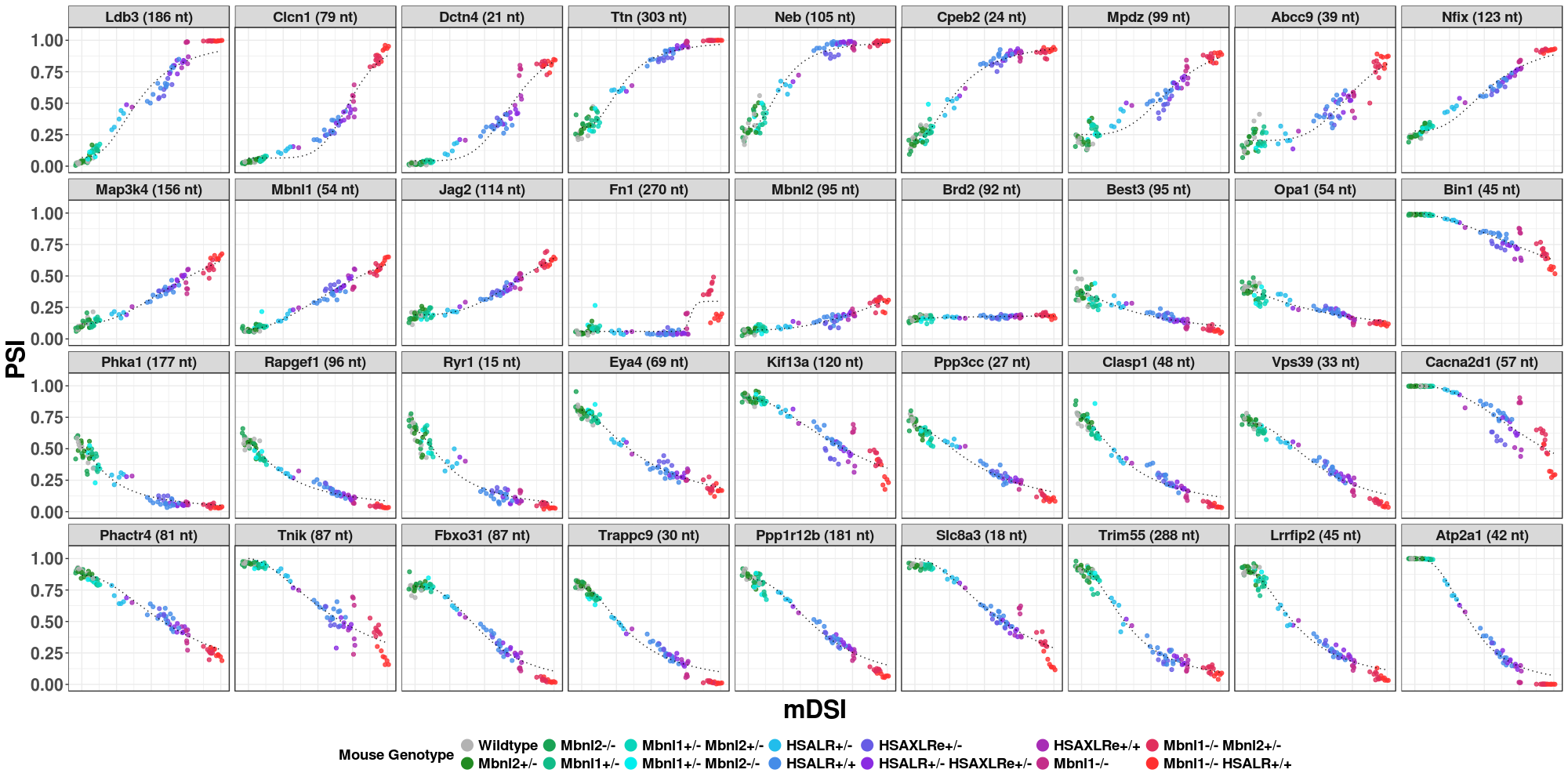


**Supplemental Figure 7. Alternative splicing quantification by targeted RNAseq for 36 events in 97 mice with varying degrees of *Mbnl* gene disruption and/or CUG^exp^ expression.**


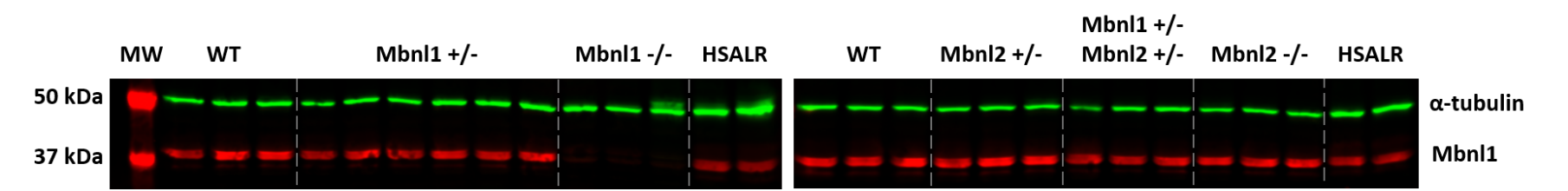


**a**


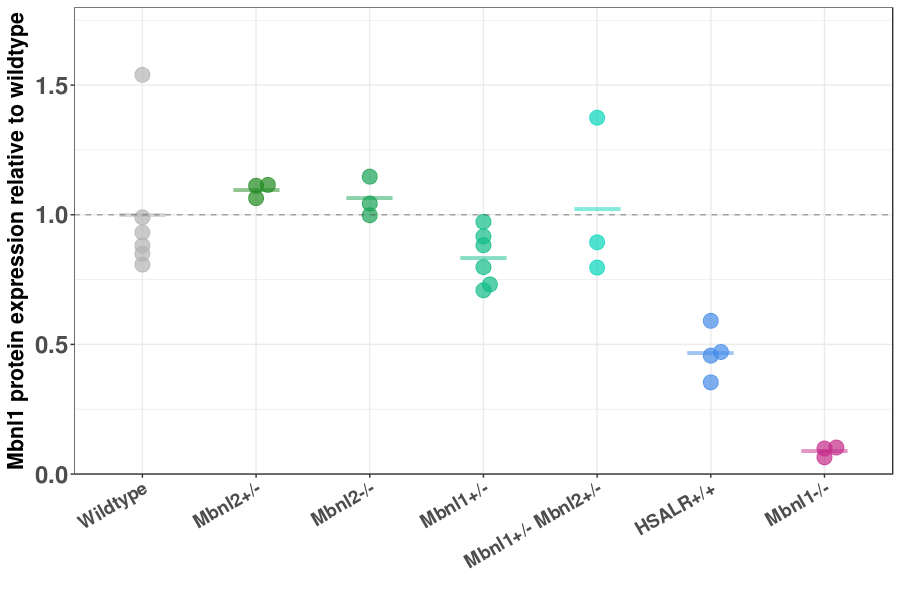


**b**

**Supplemental Figure 8. Immunoblots of Mbnl1 in quadriceps muscle from mice with *Mbnl* gene deletion or HSALR mice. *a***, immunoblots of quadriceps muscle were probed with rabbit polyclonal antibody for Mbnl1 (red) and mouse monoclonal antibody for α-tubulin as loading control (green), quantified in ***b***. Mice heterozygous for *Mbnl1* deletion have only minor reduction of Mbnl1 protein, indicating dosage compensation. Homozygous HSALR transgene mice exhibit partial reduction of Mbnl1 protein.


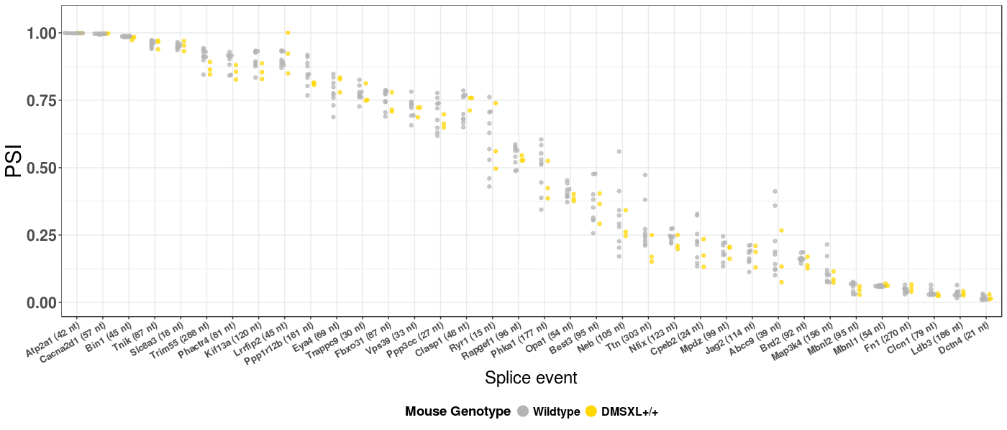


**a**

**b**


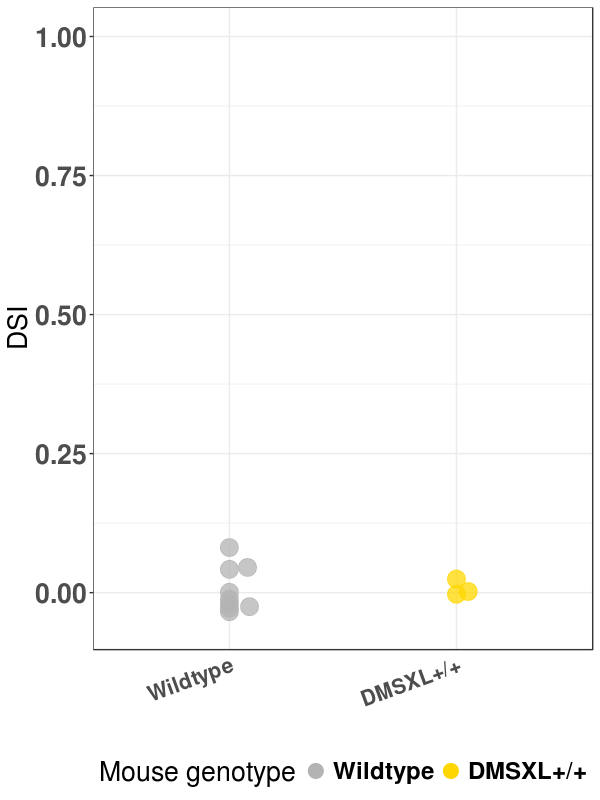


**Supplemental Figure 9**. **Targeted RNAseq of quadriceps from three homozygous DMSXL mice**. DMSXL transgenic mice express about 1200 CTG repeats from a randomly inserted cosmid containing the entire human *DMPK* gene and flanking sequences. Expression of this transgene is about 10-fold less than endogenous mouse *Dmpk* in skeletal muscle (Gudde 2016). Individual splice events (***a***) and mDSI (***b***) in DMSXL homozygotes do not differ substantially from wildtype mice.


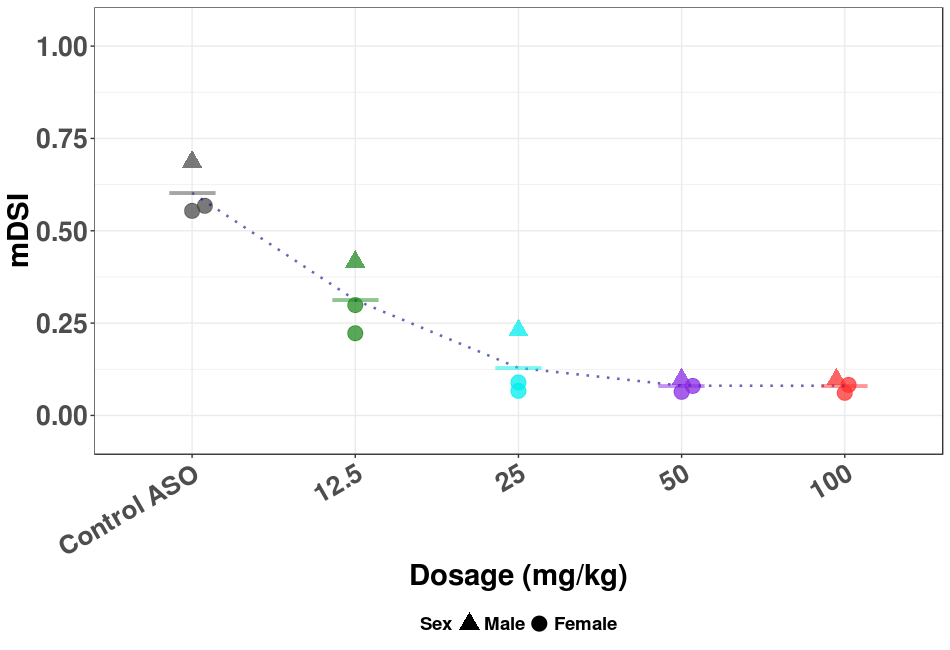

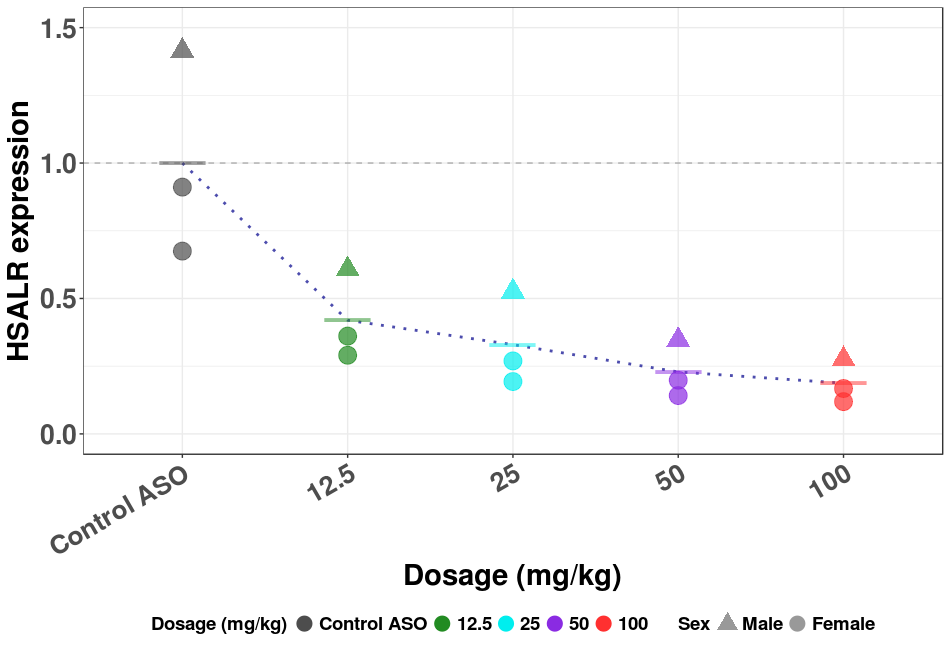


**b**

**a**


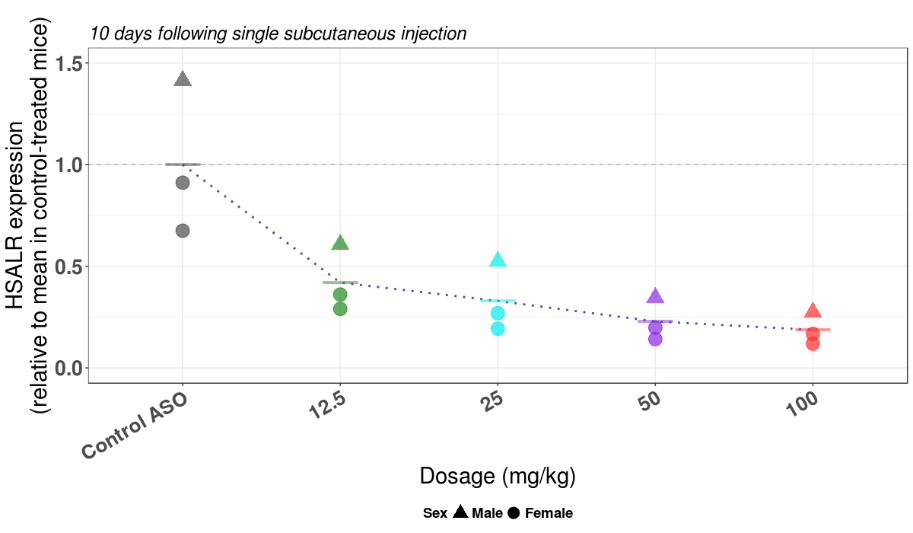


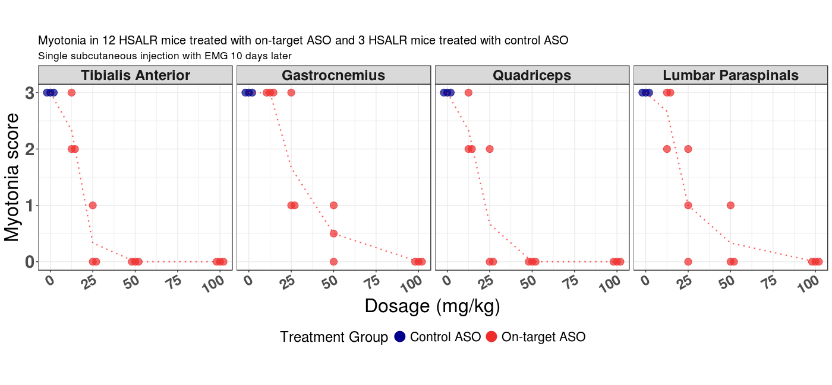


**c**

**Supplemental Figure 10**. **Dose responsivity of mouse DM1 splicing index (mDSI) to ASO knockdown of CUG^exp^ RNA.** Homozygous HSALR mice were given a single subcutaneous injection of ligand-conjugated ASO targeting *ACTA1* transgene mRNA at the indicated dose or 100 mg/kg of non-targeting ligand-conjugated ASO as control. Mice were sacrificed 10 days later for analysis. ***a***, transgene mRNA, quantified by RT-qPCR relative to *Gtf2b*, showed dose-dependent reduction as compared to non-targeting control. **b,** the correction of splicing, as reflected by mDSI, was parallel to knockdown of transgene mRNA. ***c***, EMG of the indicated muscles, performed prior to sacrifice on day 10, showed reduced myotonia at all dose levels, and complete elimination at the highest dose level. EMG was performed by blinded examiner.

**
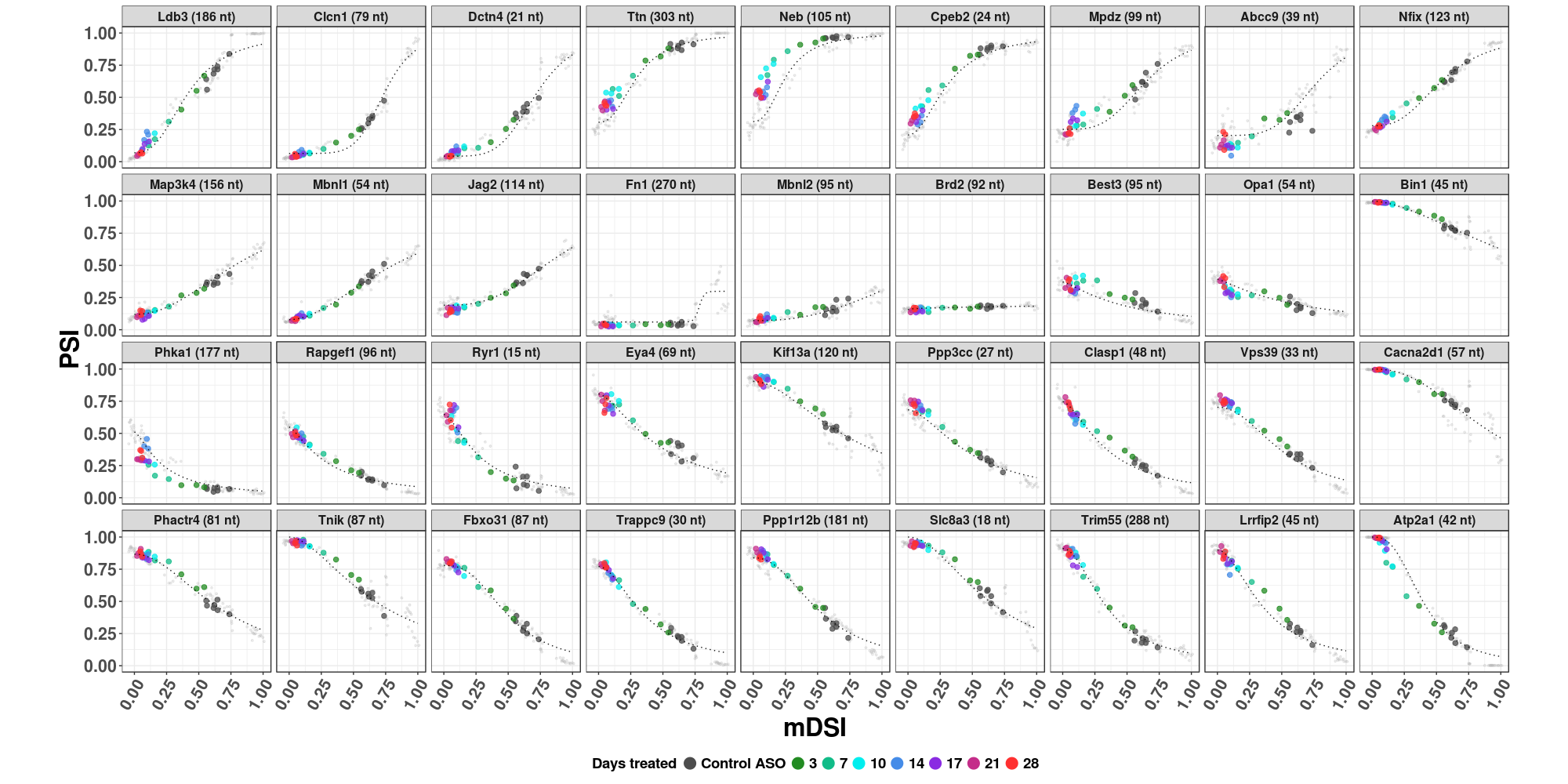
**

**a**

**
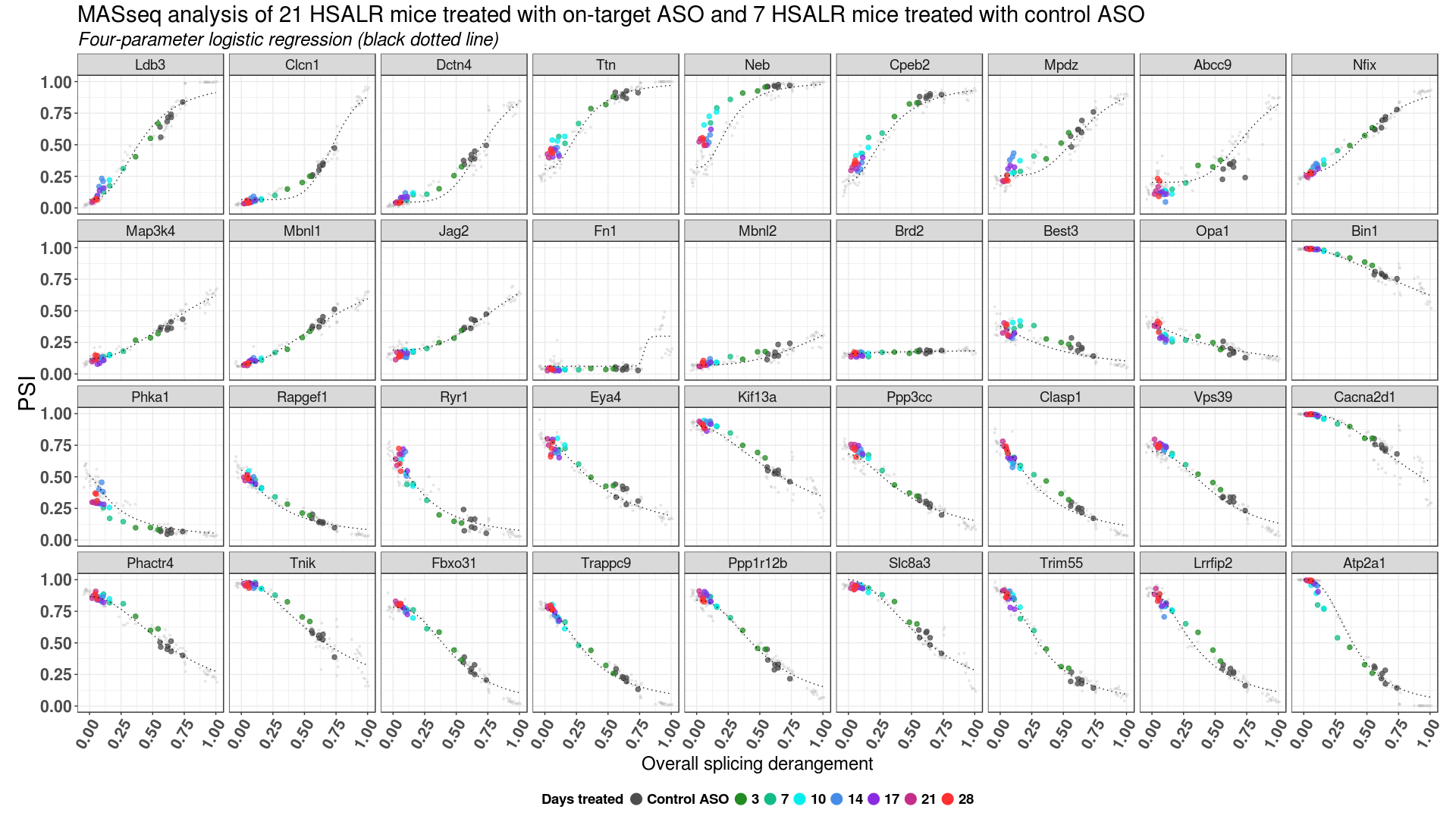
**


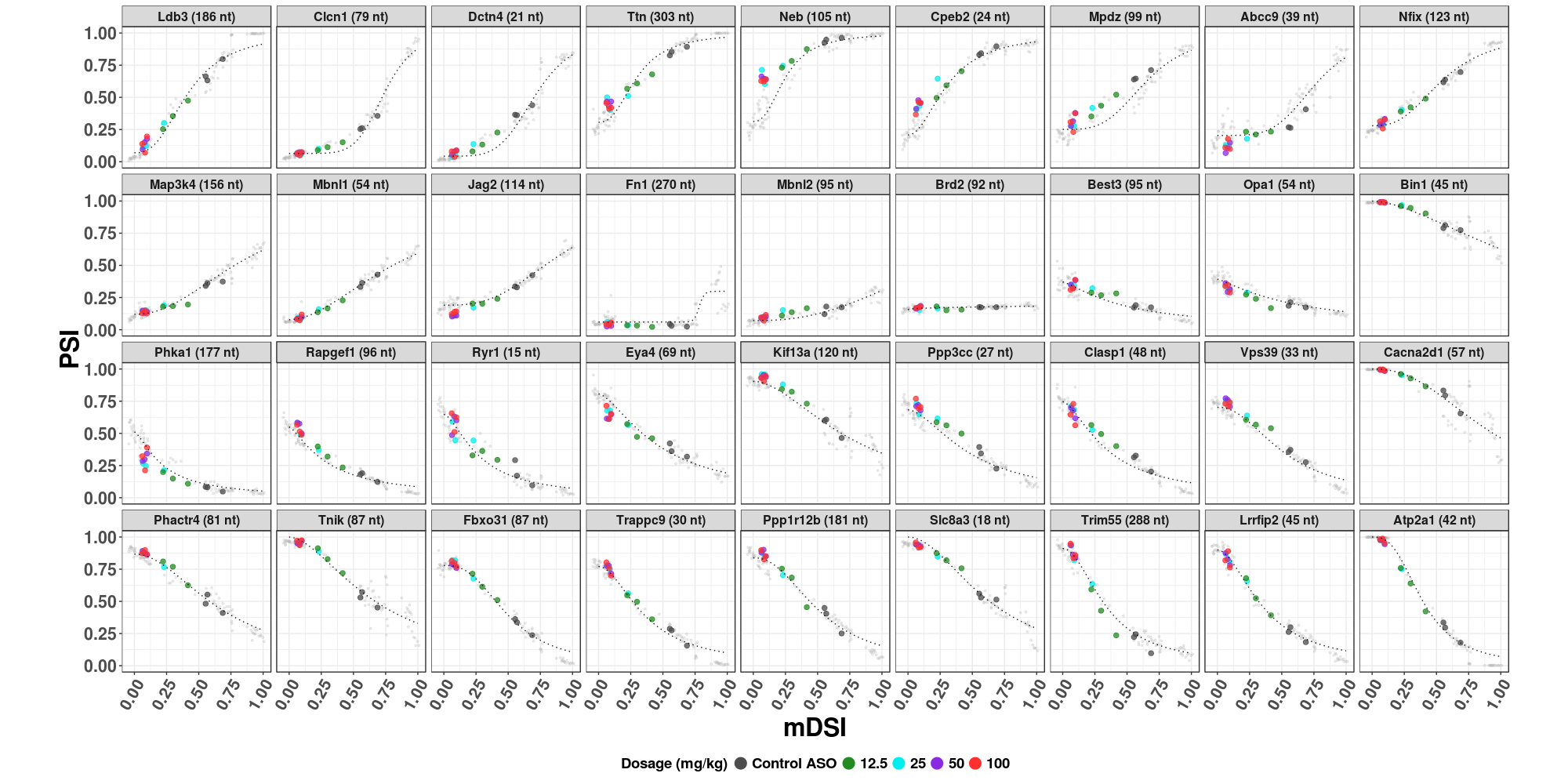


**b**


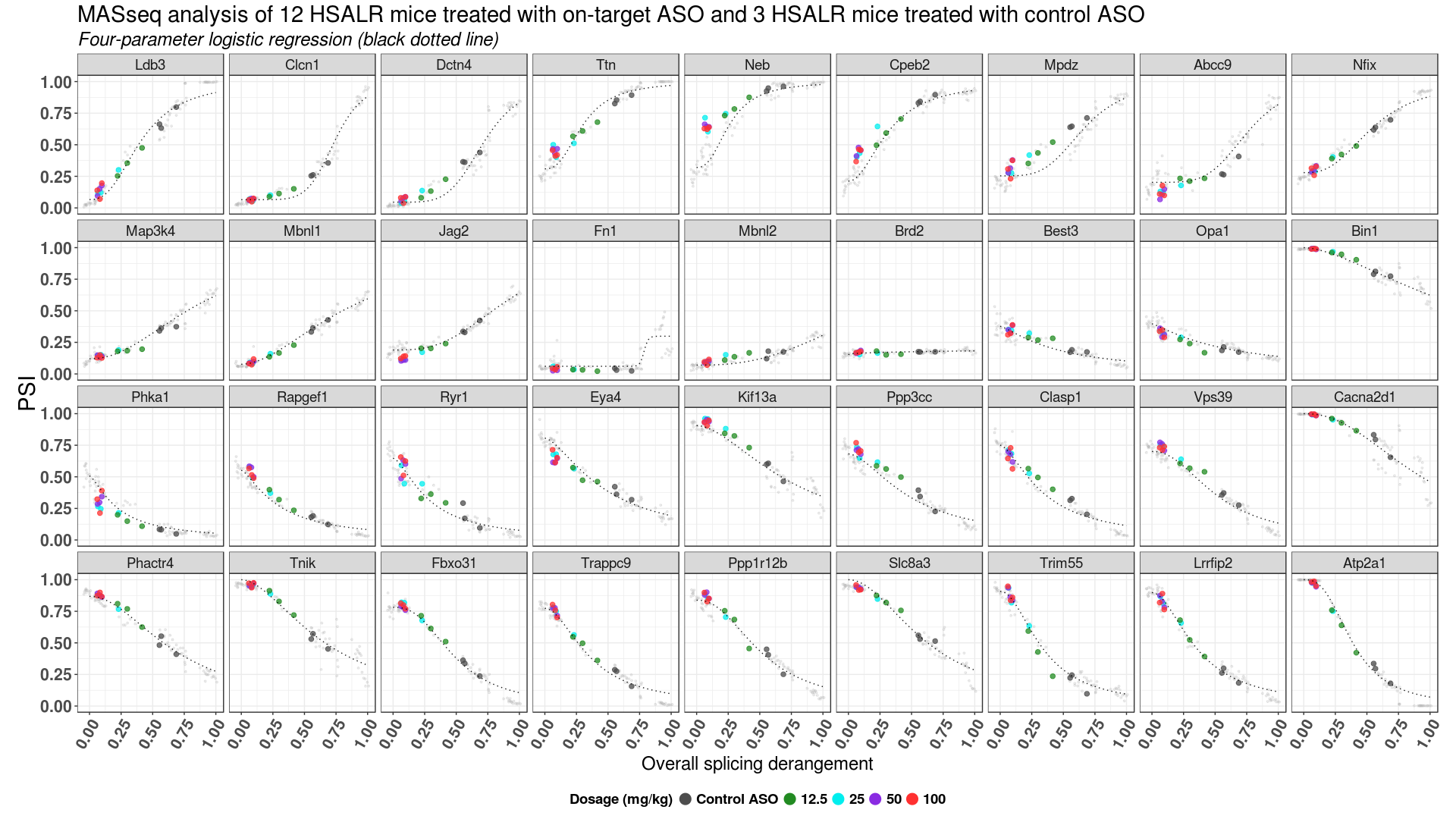


**Supplemental Figure 11.** **With few exceptions, ASO-mediated knockdown of CUG^exp^ RNA recapitulates splicing patterns observed in mice having constitutive *Mbnl* gene deletion or CUG^exp^ expression.** Shown are individual splice events plotted against overall splicing index for HSALR mice treated with multi-dose (**a**, *n* = 28) or single-dose (**b**, *n* = 15) regimens of ASOs. Small gray dots, and corresponding logistic regression lines (dotted lines), are data from Figure 5, comprising mice with various levels of constitutive *Mbnl* gene deletion or CUG^exp^ transgene expression. When splicing reverts toward normal in response to ASOs, the rescued values fall along the regression lines, indicating a specific reversion to lower levels of CUG^exp^ accumulation and higher levels of Mbnl activity. However, four DM1-affected events, *Ttn*, *Neb*, *Cpeb2*, and *Phka1*, which are also sensitive to myotonia or muscle identity, fail to resume normal splicing patterns within 10 or 28 days, indicating longer-term effects on muscle plasticity that are not rapidly reversible by Mbnl release alone.

| 998 exons mis-spliced in HSALR (\|ΔPSI\| $\boldsymbol{\geq}$ 0.05) | | | | |
| --- | --- | --- | --- | --- |
| Rank | Pentamer | Adj. *R*^2^ | Adj. *p* | Frequency |
| 1 | GCUGC | 0.023887 | 0.002252 | 0.277555 |
| 2 | GCUUC | 0.018301 | 0.019261 | 0.229459 |
| 3 | UCGCU | 0.015948 | 0.041522 | 0.05511 |
| 4 | UAAUC | 0.015413 | 0.041522 | 0.108216 |
| 5 | CCGCU | 0.014642 | 0.049045 | 0.08016 |
| 6 | CUAAU | 0.013139 | 0.087227 | 0.143287 |
| 7 | CGCUA | 0.012284 | 0.102876 | 0.023046 |
| 8 | UGCGC | 0.012241 | 0.102876 | 0.052104 |
| 9 | GCUUG | 0.0119 | 0.108528 | 0.185371 |
| 10 | UGGCU | 0.011005 | 0.143092 | 0.272545 |

**Supplemental Table 1. Analysis of motifs in flanking sequences reveals pentamers that are most predictive of misregulated splicing in HSALR mice.** For all pentamers, a linear model was constructed using upstream and downstream occurrences as explanatory variables and ΔPSI as the outcome variable across all mis-spliced exons in HSALR mice (|ΔPSI|$\geq$0.05 compared to WT, FDR-adjusted *p* < 0.05, and at least 10 isoform-specific counts). Filtering for pentamers with high inter-species sequence conversation was performed as described in Methods. The flanking sequences for this analysis included 200 nt of flanking intron and 50 nt of exon adjacent to 5′ and 3′ splice sites. All pentamers were ranked by dose- and position-dependent (up or downstream of exon) capacity to explain splicing outcomes, given by *R*^2^. *P* values were determined by F-test and adjusted for multiple testing using the Benjamini-Hochberg method. “Frequency” denotes the fraction of flanking regions that contained the listed pentamer. Among the 10 top-ranked pentamers listed, 8 were putative Mbnl-binding sites, and #4 and #6 were putative binding sites for Quaking, a factor previously implicated in splicing regulation in muscle cells (Hall 2013).

| 1,498 exons mis-spliced in Mbnl¾KO (\|ΔPSI\| $\boldsymbol{\geq}$ 0.05) | | | | |
| --- | --- | --- | --- | --- |
| Rank | Pentamer | Adj. *R*^2^ | Adj. *p* | Frequency |
| 1 | GCUGC | 0.017577 | 0.000666 | 0.283901 |
| 2 | UCUGC | 0.012224 | 0.016495 | 0.270541 |
| 3 | UGGCU | 0.011812 | 0.016495 | 0.269873 |
| 4 | CUGCU | 0.011514 | 0.016495 | 0.365397 |
| 5 | GCUUU | 0.010684 | 0.024701 | 0.297929 |
| 6 | UGCCC | 0.010238 | 0.028821 | 0.177021 |
| 7 | UCGCU | 0.009868 | 0.032654 | 0.048764 |
| 8 | UGCUU | 0.009055 | 0.048891 | 0.336673 |
| 9 | CUAAU | 0.008956 | 0.048891 | 0.147629 |
| 10 | CGCUG | 0.008809 | 0.048891 | 0.079492 |

**Supplemental Table 2. Pentamer motifs with greatest capacity to explain Mbn*l*¾KO mis-splicing.** As in Supplemental Table 1, except that analysis was carried out on 1,498 exons mis-spliced in Mbnl¾KO mice. The top-ranked pentamers were all putative Mbnl-binding sites, except for #9, a putative binding site for Quaking.

**a**

| **GO Biological Process (Mbnl¾KO log2foldchange > 0.5)** | **Overlap** | **Adjusted *p*** |
| --- | --- | --- |
| extracellular matrix organization (GO:0030198) | 66/229 | 2.58E-11 |
| platelet degranulation (GO:0002576) | 37/124 | 4.25E-06 |
| regulation of cell migration (GO:0030334) | 68/316 | 6.16E-06 |
| regulated exocytosis (GO:0045055) | 39/148 | 3.70E-05 |
| cellular response to cytokine stimulus (GO:0071345) | 84/456 | 9.69E-05 |
| actin filament organization (GO:0007015) | 33/120 | 1.13E-04 |
| regulation of apoptotic process (GO:0042981) | 130/815 | 2.13E-04 |
| muscle contraction (GO:0006936) | 35/137 | 2.41E-04 |
| negative regulation of apoptotic process (GO:0043066) | 86/485 | 2.53E-04 |

**b**

| **GO Biological Process (Mbnl¾KO log2foldchange < -0.5)** | **Overlap** | **Adjusted *p*** |
| --- | --- | --- |
| muscle contraction (GO:0006936) | 18/137 | 2.99E-05 |
| glucan catabolic process (GO:0009251) | 7/18 | 3.00E-04 |
| glycogen catabolic process (GO:0005980) | 7/18 | 2.00E-04 |
| actomyosin structure organization (GO:0031032) | 12/71 | 1.58E-04 |
| myofibril assembly (GO:0030239) | 10/47 | 1.49E-04 |
| cellular polysaccharide catabolic process (GO:0044247) | 7/19 | 1.55E-04 |
| positive regulation of ryanodine-sensitive calcium-release channel activity (GO:0060316) | 5/8 | 3.13E-04 |
| positive regulation of calcium ion transmembrane transporter activity (GO:1901021) | 6/17 | 0.001225387 |
| muscle filament sliding (GO:0030049) | 8/38 | 0.001625625 |

**Supplemental Table 3**. Gene ontology enrichment analysis revealed functional categories that showed upregulated (**a**) or downregulated (**b**) gene expression in Mbnl¾KO mice.

| **Transcription factor-binding motif (Mbnl**¾**KO log2foldchange < -0.5)** | **Overlap** | **Adjusted p** |
| --- | --- | --- |
| CTAWWWATA V$RSRFC4 Q2 | 25/370 | 0.00262834 |
| V$MEF2 02 | 17/238 | 0.022669561 |
| V$RSRFC4 01 | 17/251 | 0.028892732 |
| V$RSRFC4 Q2 | 15/219 | 0.046703446 |
| V$MYOD Q6 | 15/254 | 0.17202833 |
| V$E12 Q6 | 15/270 | 0.259324237 |
| V$SRF Q6 | 14/249 | 0.273904821 |
| V$AR 03 | 6/60 | 0.248292183 |
| V$MEF2 Q6 01 | 14/251 | 0.228859088 |

**Supplemental Table 4**. Among promoter regions of genes downregulated in Mbnl¾KO mice, analysis for transcription factor recognition motifs showed significant enrichment for Mef2 transcription factors. RSRFC4 is an alternate name for Mef2a, which shares a DNA-binding motif with Mef2c and Mef2d. Analysis of promoter region position weight matrices (PWMs) performed using Enrichr with UCSC Genome Browser PWM database.
